# Supplementary material for: Non-equilibrium steady states in supramolecular polymerization
Source: Nat Commun. 2017 Jun 19;8:15899. doi: 10.1038/ncomms15899 (PMC5481825; doi:10.1038/ncomms15899)
Supplement: Supplementary Information [file ncomms15899-s1.pdf]

Type of file: PDF
Size of file: 0 KB
Title of file for HTML: Supplementary Information
Description: Supplementary Figures, Supplementary Tables, Supplementary Discussion, Supplementary Methods and Supplementary References

Type of file: PDF
Size of file: 0 KB
Title of file for HTML: Peer Review File
Description:

## Supplementary Methods

### Materials

Fmoc-proteced α-L-amino acids and the 2-chlorotrityl resin loaded with L-Leu (200-400 mesh) were purchase from BACHEM. ATP was purchased from Sigma Aldrich. The other chemicals were purchased from Sigma Aldrich, TCI and Carl Roth, and used as received. The enzymes Protein Kinase A, PKA, Catalytic Subunit (13 μM solution in Tris-HCl buffer/glycerol), Alkaline Phosphatase, Calf Intestine, CIP, (48 μM solution in Tris-HCl buffer/glycerol) and Lambda Protein Phosphatase, λPP, (20 μM solution in HEPES buffer/glycerol) were obtained from New England BioLabs.

UV-Vis spectra were recorded using a JASCO V-670 spectrophotometer equipped with a Peltier system as temperature controller. When not differently specified, spectra were recorded in a 1 mm quartz cuvette.

CD spectra were recorded on JASCO J-1500 and a JASCO J-810 spectropolarimeters equipped with a Peltier system as temperature controller. When not differently specified, spectra were recorded in a 1 mm quartz cuvette.

LC-MS was performed with an Accela HPLC (Thermo Fisher Scientific; Hypersil GOLDcolumn, 50 x 2.1 mm, 1.9 μm) integrated with an LCQ Fleet ion-trap (Thermo Fisher Scientific).

Atomic force microscopy (AFM) measurements were performed under ambient condition using a Bruker Veeco Dimension 3100 AFM microscope with Nanoscope IV controller, operating in tapping mode. Silica probes Bruker TESPAW-V2 with a resonance frequency of ~360 kHz and a spring constant of ~80 $Nm^{-1}$ were used.

TEM Images were recorded using a Philips CM120 Transmission Electron Microscope operating at 100 kV with a LaB6 filament equipped with a Peltier cooled slow scan CCD camera (Model 794, Gatan, Pleasanton).

## Synthesis

### *Synthesis of the perylene-L-alanine core, [N,N'-di(L-alanine)-perylene-3,4:9,10-tetracarboxylic acid diimide], 1*

Perylene-3,4,9,10-tetracarboxylic acid bisanhydride (700 mg, 1.78 mmol), L-alanine (380 mg, 4.27 mmol, 2.4 eq) and imidazole (5 g, 73 mmol, 41 eq) were purged with $N_2$ in a 250 mL double neck round-bottom flask for 15 min. Then, the reaction mixture was heated up to 130 ºC and left stirring 30 min under $N_2$ atm. Afterwards, the mixture was cooled down to 90 ºC and 100 mL of water was added. The resulting solution was filtered and the filtrate was added dropwise to 300 mL of isopropanol giving a red precipitate. The red solid was collected by filtration and dried under vacuum affording compound **1** (800 mg, 85 %). $^1$H NMR (400 MHz, DMSO-d6): δ 8.07 (s, 8H), δ 5.56 (dd, *J* = 7.0 Hz, 2H), 1.68 (d, *J* = 7.0 Hz, 6H).

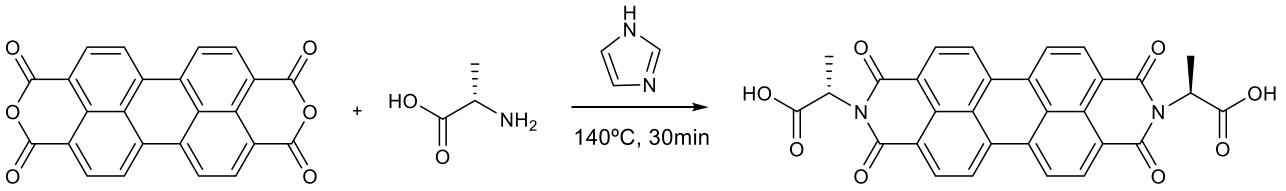


**Supplementary Figure 1 |** Preparation of the perylene-L-alanine core

### *Synthesis of Fmoc-LR(Pbf)R(Pbf)AS(tBu)L-OH peptide, 2*

Peptide **2** was prepared by manual Fmoc solid phase peptide synthesis, SPPS (Supplementary Figure 2), starting from 2-chlorotrityl resin loaded with L-leucine (200-400 mesh, 0.79 mmol/g capacity, typically 1 g). This is a highly acid labile resin that requires only mild conditions for the final cleavage, which allowed to obtain fully protected **2** (i.e. Fmoc protected at the N-terminus, as well as at the sensitive side chains). The typical protocol involved: a) swelling of the resin (DMF); b) activation of the new amino acid at the carboxylate (with HBTU, 1 eq., HOBt, 1 eq., in the presence of DIPEA, 2 eq. for 10 min); c) coupling reaction (3 eq. of activated amino acid with respect to resin capacity) for 50 min followed by 10 washes with DMF to get rid of the excess of reactants; d) Fmoc deprotection to liberate the *N*-terminus of the growing peptide (piperidine/DMF 1:4, 2 x 20 min) plus 10 washes with DMF. The synthesis cycle b – d was repeated for the different amino acids (Fmoc deprotection was not done at the last cycle). The activation step b was not performed in the case of arginine, to avoid the possible formation of an intermolecular δ-lactame that would decrease the coupling yield.[1] Finally, the fully protected target peptide **2** was cleaved off the resin by treatment with 1% TFA in dichloromethane for 1h. Then **2** was dissolved in dichloromethane and washed with 1 M aqueous HCl to remove traces of piperidine coming from the SPPS. The latter was a necessary step in order to avoid the formation of unwanted acyl-piperidine derivatives in the next coupling reactions. HRMS (ESI+) Calculated Mass for $C_{75}H_{109}N_{12}O_{16}S_2$ [M+H$^+$]: 1497.7520. Found: 1497.7368.

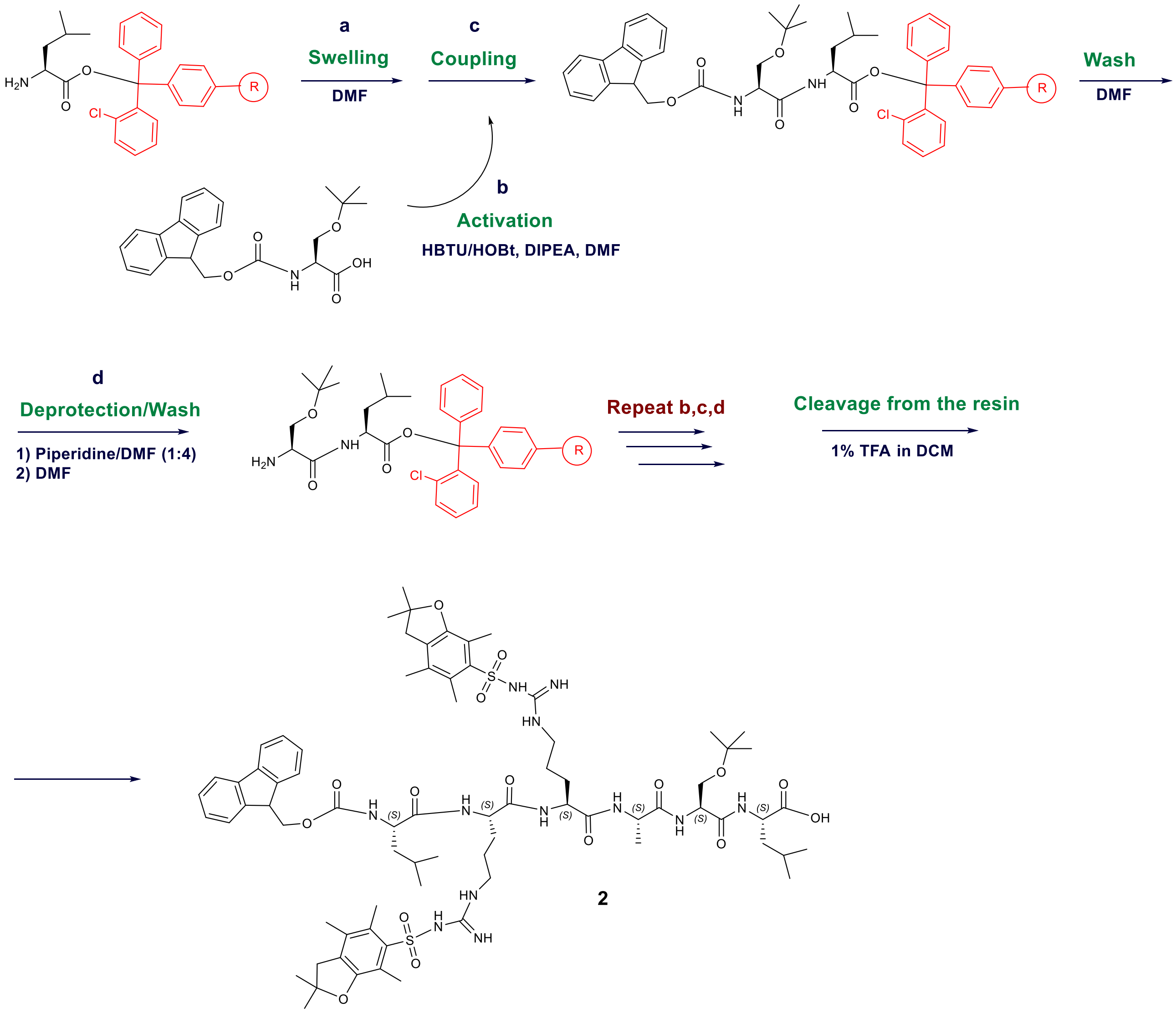


**Supplementary Figure 2 |** Fmoc solid phase peptide synthesis using the highly acid labile 2-chlorotrityl resin. The fully protected peptide Fmoc-LR(Pbf)R(Pbf)AS(tBu)L-OH was obtained with yields > 90%.

### *Synthesis of H- LR(Pbf)R(Pbf)AS(tBu)L-TEG, 5*

HBTU (137 mg, 0.36 mmol, 1.2 eq) and DIPEA (157 µL, 0.9 mmol, 3 eq) were added to a solution of peptide **2** (450 mg, 0.3 mmol, 1 eq) in 8 mL DMF under $N_2$ atm. After 5 min, TEG-amine **3** (58.9 mg, 0.36 mmol, 1.2 eq) dissolved in 4 mL DMF was added to this solution and the mixture left stirring overnight (compound **3** was prepared according to a reported procedure[2]). Then, the reaction was quenched by the addition of 100 mL dichloromethane, and the organic phase was washed with saturated $NaHCO_3$ and brine, dried over anhydrous $MgSO_4$ and concentrated to give **4**. Afterwards, the Fmoc protecting group was removed by treating compound **4** with 5 mL of a piperidine/DMF 1:4 mixture during 30 min. After this time, the solvent was evaporated and the crude re-dissolved in 100 mL MeOH. The obtained solution was washed with heptane (5x100 mL) and concentrate to afford **5** (380 mg, 89% yield) as a sticky solid. HRMS (ESI+) Calculated Mass for $C_{67}H_{114}N_{13}O_{16}S_2$ [M+H$^+$]: 1420.7942. Found: 1420.7786.

## *Synthesis of target PDI*

HBTU (64 mg, 0.17 mmol, 2.4 eq) and DIPEA (125 µL, 0.72 mmol, 9 eq) were added to a solution of **1** (38 mg, 0.07 mmol, 1 eq) in 8 mL DMF under $N_2$ atm. After 5 min, **5** (220 mg, 0.15 mmol, 2.2 eq) dissolved in 4 mL DMF was added and the reaction mixture was left stirring overnight. The reaction was quenched by the addition of 50 mL dichloromethane, and the organic phase was washed with saturated $NaHCO_3$ and brine, dried over anhydrous $MgSO_4$ and concentrated. The reaction crude was purified by flash chromatography using $CH_2Cl_2$/MeOH (gradient from 100:0 to 80:20) as eluent to give **6.** Finally, the deprotection of the side chain protecting groups were carried out under strong acid conditions by treatment with TFA/$H_2O$, 9/1(4 mL) for 4h. The latter solution was poured into 70 mL of diethyl ether resulting in the precipitation of a red solid, that was filtered, washed with diethyl ether, and dried affording **PDI** (75 mg, 40%). $^1$H NMR (400 MHz, $D_2O$) δ 8.14 (m, 6H), 5.65 (s, 2H), 4.36 (m, 10H), 4.00 – 3.51 (m, 24H), 3.38 (m, 18H), 1.62 (m, 48H), 0.91 (m, 28H), 0.46 (s, 4H). HRMS (ESI$^+$) for $C_{104}H_{164}N_{28}O_{26}$ [M+4H$^+$]: calculated 555.3087; found: 555.3115. [M+3H$^+$]: calculated 740.0759; found: 740.0778. [M+2H$^+$]: calculated 1109.6102; found: 1109.6053.

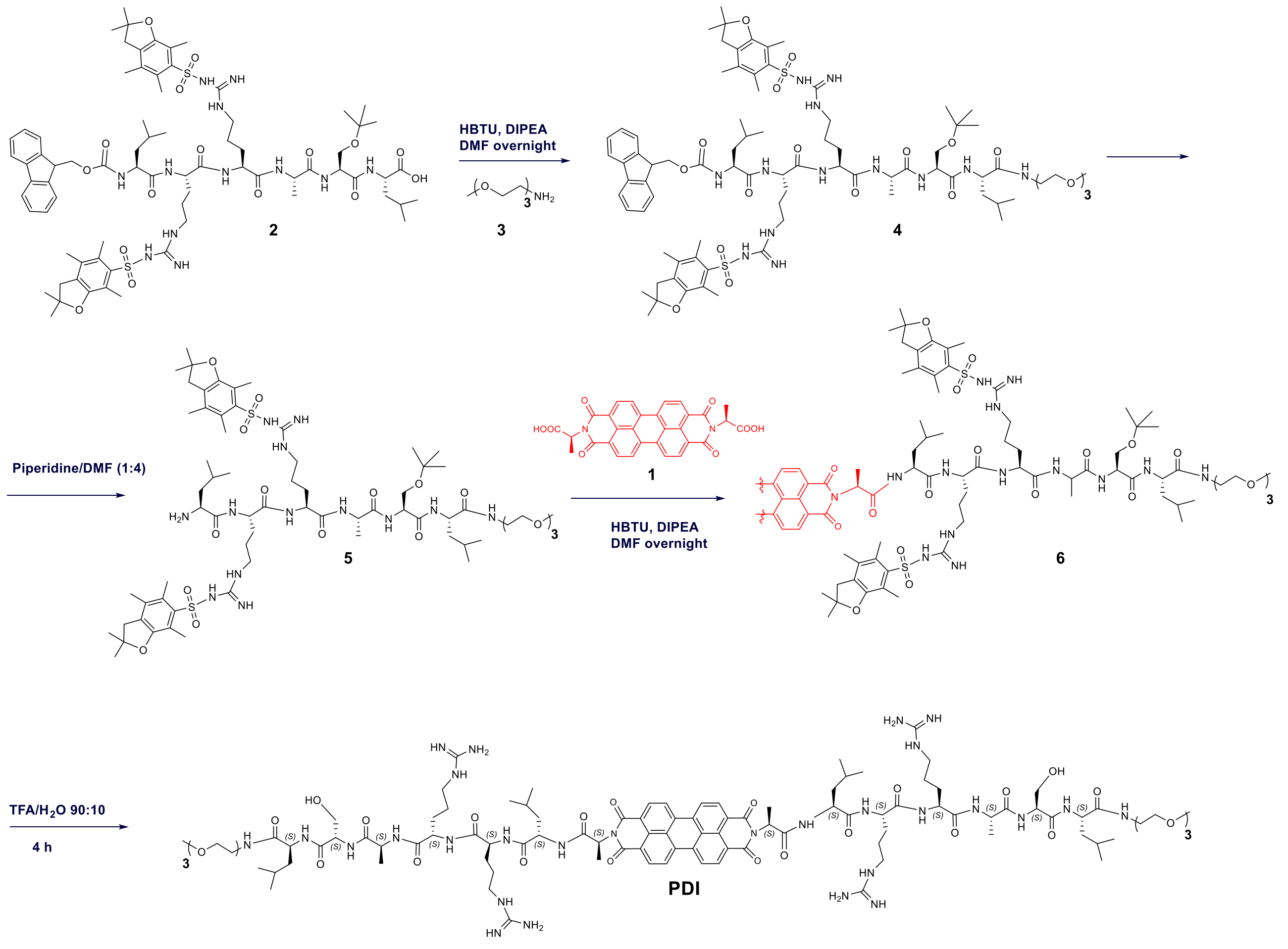


**Supplementary Figure 3 |** Preparation of the final target **PDI.**

## Supplementary Discussion

### Overview of the enzyme controlled supramolecular polymerization

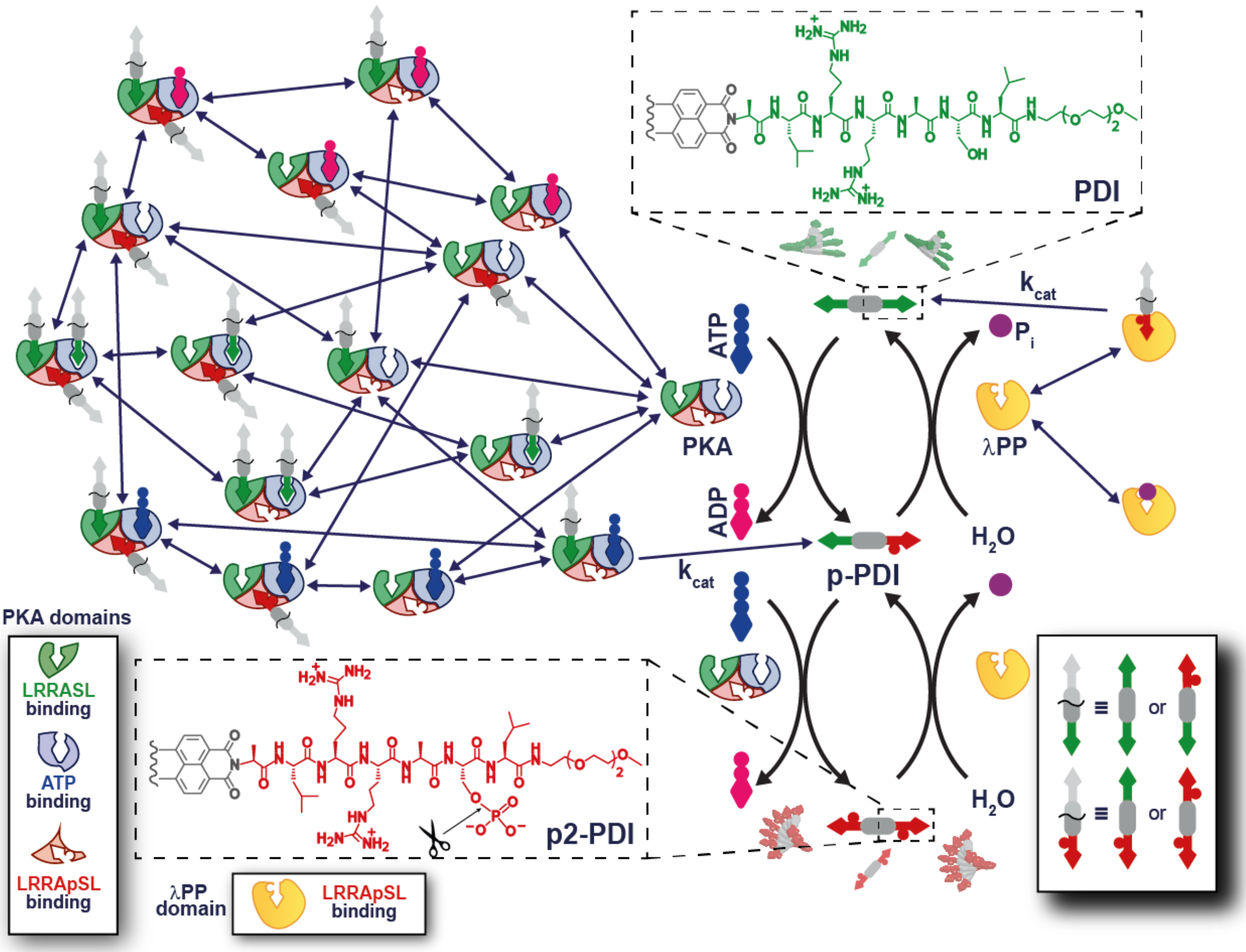


**Supplementary Figure 4 |** General overview of the biomimetic enzyme-controlled supramolecular system. The self-assembling peptide-perylenediimide derivative **PDI** (half molecule is shown) can be phosphorylated on the serine residue by Protein Kinase A (PKA) to give monophosphorylated **p-PDI**, and further diphosphorylated **p2-PDI**, fueled by ATP to ADP hydrolysis (one eq. per phosphate introduced). Diphosphorylated **p2-PDI** can in turn be dephosphorylated back to give **PDI** (scissors) by Lambda Protein Phosphatase (λPP), which yields inorganic phosphate Pi as waste. The two enzymes work simultaneously, so that the chemical composition of the solution (molar fraction of **PDI**, **p-PDI**, and **p2-PDI**) is determined by the rates of competing phosphorylation and dephosphorylation pathways, but not by chemical equilibrium. This allowed us to push the system into dissipative non-equilibrium steady states (NESS), when working in a membrane reactor, i.e. open system (cfr. Fig 4 main text). A schematic representation of the enzyme network(s) is reported. The nodes in the PKA (and λPP) network represent the enzyme-substrate complexes. The edges (double-headed arrows) represent the binding reactions. PKA has three binding sites: i) for ATP (blue), ii) for the LRRASL peptide (green), and iii) for LRRApSL (red). Both **PDI** and **p2-PDI** self-assemble to give supramolecular polymers with different structure and stability (see main text).

## Studying of phosphorylation/dephosphorylation

### *Phosphorylation of PDI using PKA*

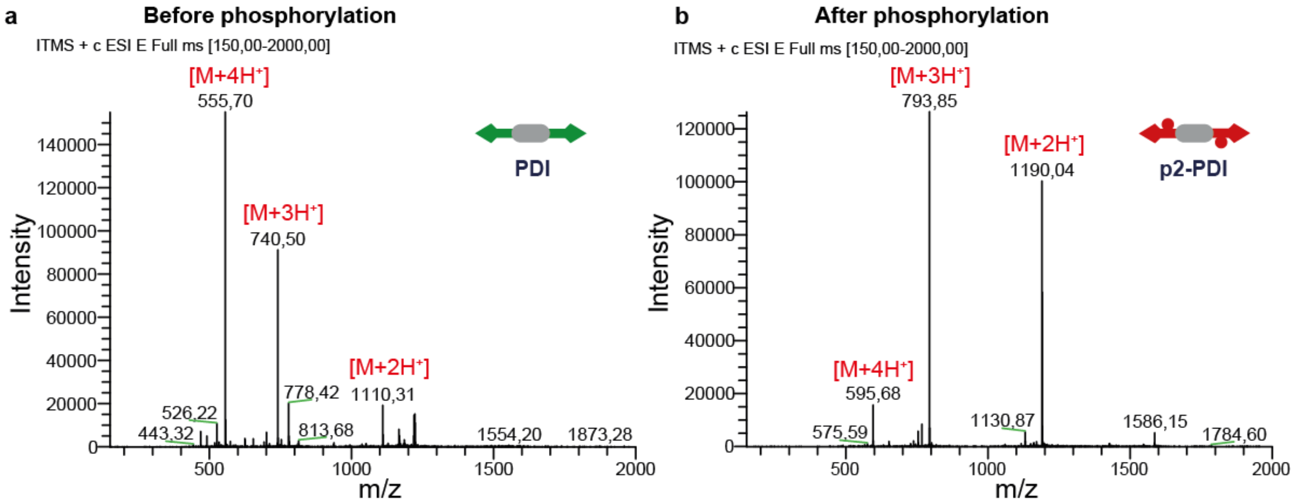


**Supplementary Figure 5 |** LC-MS spectra of a 1 mM **PDI** solution (injection: 4 µM) in the presence of ATP (2 mM, 2eq.), before phosphorylation (**a**), and after complete phosphorylation to **p2-PDI** (~ 4h) triggered by the addition of 0.13 µM PKA (**b**).

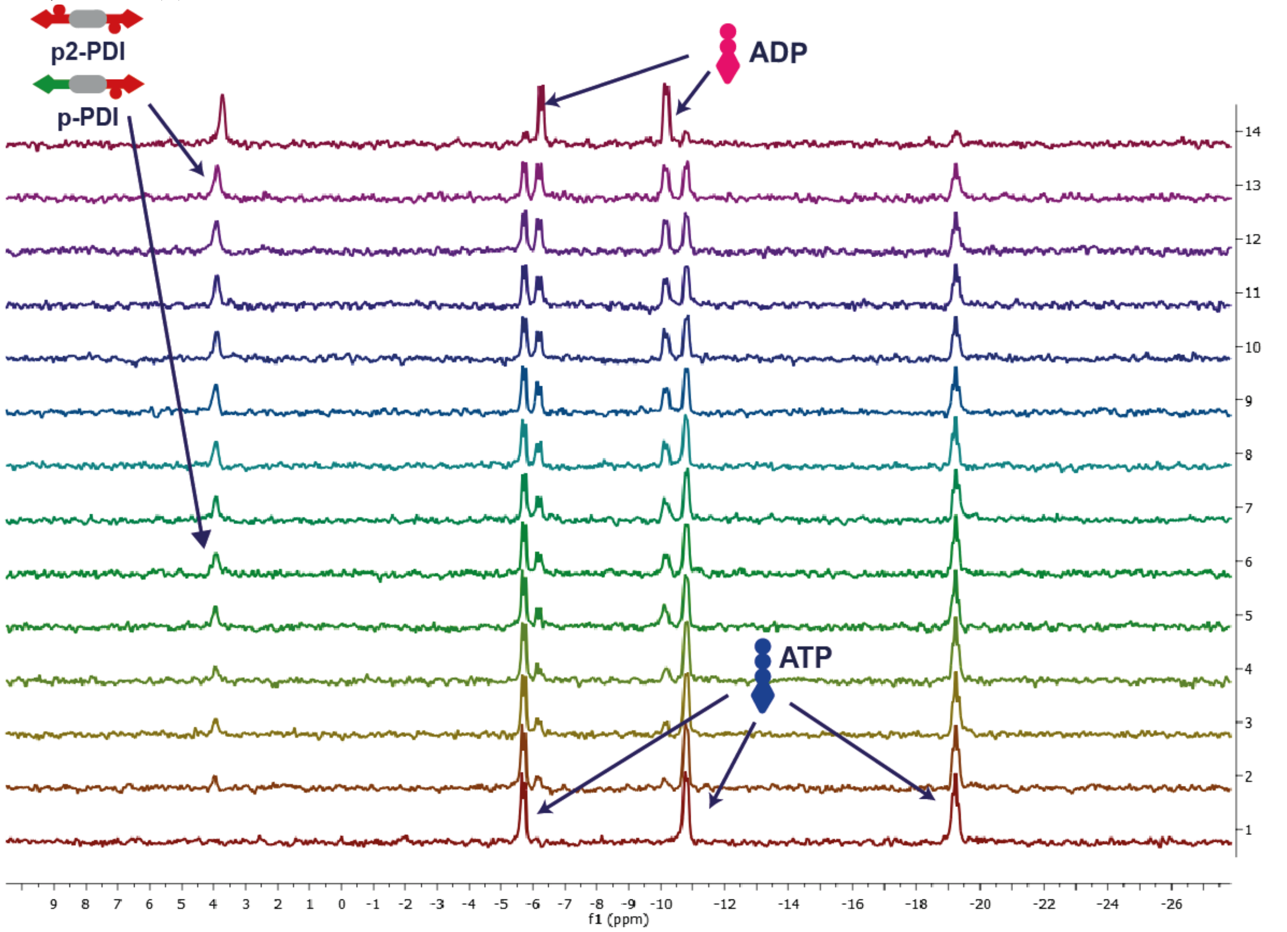


**Supplementary Figure 6 |** $^{31}$P-NMR spectra recorded each 10 min during the phosphorylation of a 0.5 mM **PDI** solution in Tris buffer (+ 20% $D_2O$) in the presence of ATP (1 mM, 2 eq.), triggered by the addition of PKA (0.04 µM). The peak of phosphoserine (LRRApSL in both **p-PDI**, and **p2-PDI**) increases with time, along with the clear conversion of ATP to ADP.

### *Dephosphorylation of p2-PDI using λPP*

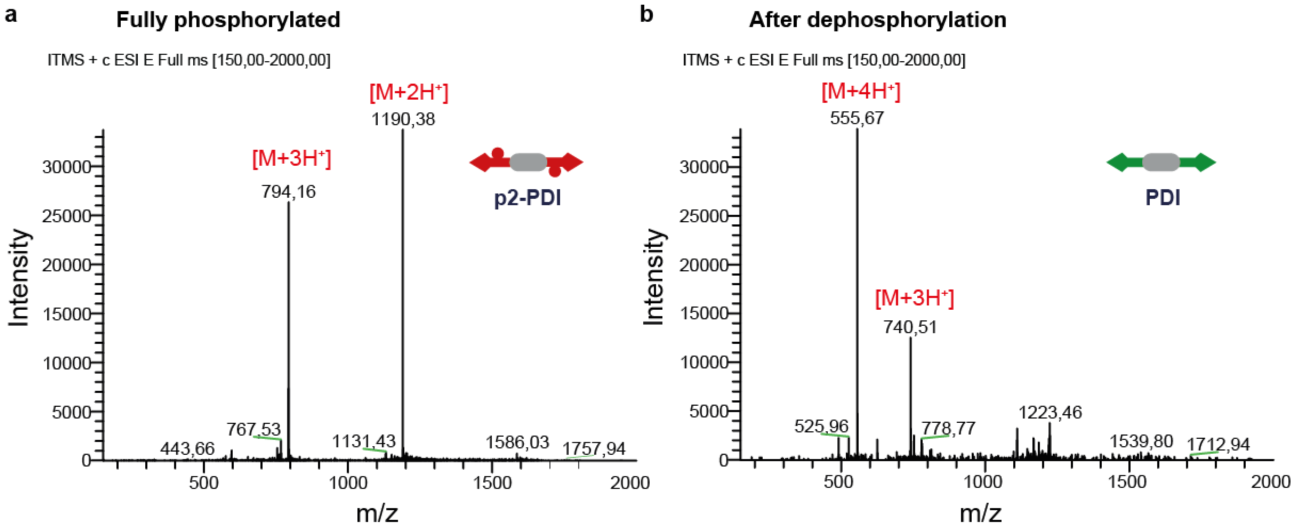


**Supplementary Figure 7 |** LC-MS spectra of 200 μM **p2-PDI** solution prepared by phosphorylating **PDI** with ATP (400 μM, 2 eq.) and PKA (0.065 μM) (**a**), and after complete dephosphorylation back to **PDI** triggered by the addition of λPP (0.065 μM) (**b**).

### *Previous attempts to dephosphorylate using Alkaline Phosphatase (Calf Intestine, CIP)*

Although phosphatases are generally marked as non-specific, we found that Alkaline Phosphatase (Calf Intestine, CIP), our first choice, is completely inactive on **p2-PDI**. In order to shed light on the molecular features at the basis of such inactivity, we prepared the model non-assembling peptide H-LRRASL-TEG ($M_1$), i.e, with the same structure as the **PDI** arms, and we studied its phosphorylation/dephosphorylation using as a comparison the commercial Kemptide H-LRRASLG-OH ($M_2$). While both peptides could be quantitatively phosphorylated by adding PKA to their 1:1 mixture in the presence of 1 eq. of ATP (Supplementary Figure 8a). The following addition of CIP (1.4 μM) resulted in the dephosphorylation of only H-LRRApSLG-OH ($pM_2$), while H-LRRApSL-TEG remained unreacted in solution even after days (Supplementary Figure 8b). The latter clearly demonstrates that the TEG tail inhibits CIP.

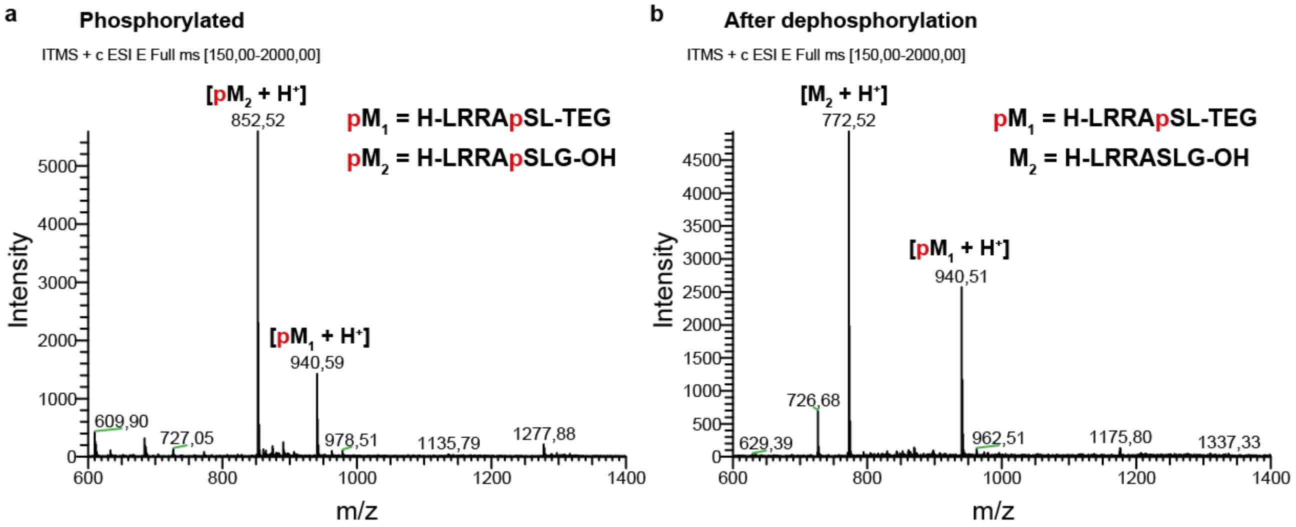


**Supplementary Figure 8 | a,** LC-MS spectrum of a 200 μM 1:1 mixture of H-LRRApSL-TEG ($pM_1$) and H-LRRApSLG-OH ($pM_2$) prepared by phosphorylation with ATP (200 μM, 1 eq) and PKA (0.065 μM). **b,** LC-MS spectrum of the same solution after the addition of CIP (1.4 μM), and incubation 90 min, showing the complete dephosphorylation of only $pM_2$.

## UV-Vis spectra and study of the supramolecular polymerization

### *UV-Vis spectrum of monomeric PDI*

As described in the main text **PDI** is strongly assembled at the investigated concentrations (0.2-1 mM) in the reaction buffer, at 25°C. However, we could obtain complete disassembly by heating a diluted (5 μM) **PDI** solution to 85°C, as shown by the UV-Vis spectrum in Supplementary Figure 9. In the latter a well-resolved vibronic structure with a ratio between the two maxima ($A_{504nm}/A_{540nm}$) of 0.65 is visible, which is characteristic of monomeric perylenes.[3,4] No additional spectral changes were observed upon further dilution and/or heating. Note that upon disassembly a slight blue shift of the two maxima (to 497 and 533 nm) also occurs, nevertheless we will continue to refer to the ratio of their absorbances as to $A_{504nm}/A_{540nm}$.

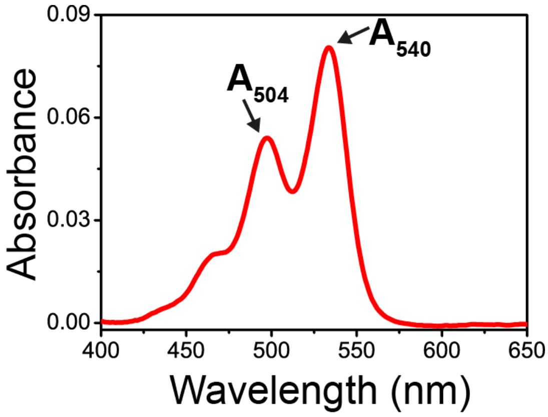


**Supplementary Figure 9 |** UV-Vis spectrum of a 5 μM **PDI** solution in the reaction buffer at 85°C. Optical path: 10 mm.

### *Thermodynamics of supramolecular polymerization*

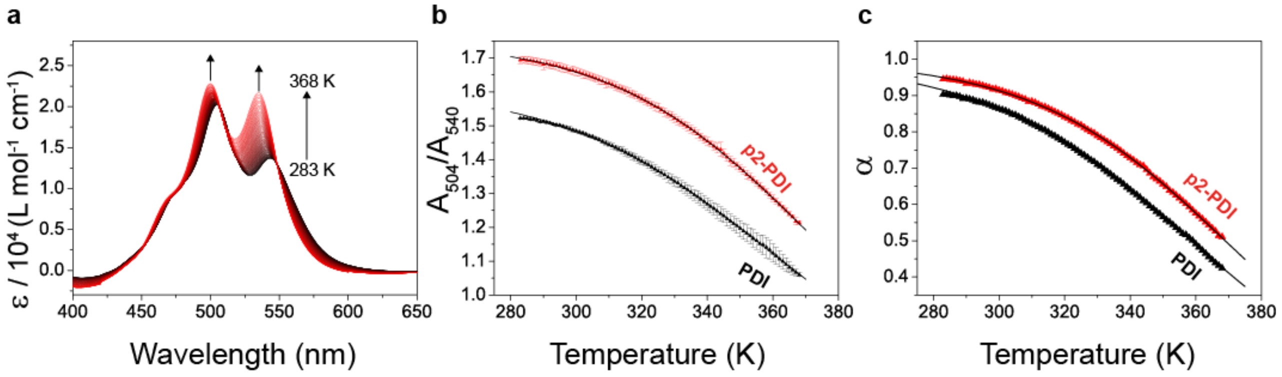


**Supplementary Figure 10 | a,** Temperature dependent UV-Vis spectra of a 230 μM **PDI** solution in the reaction buffer and in the presence of ATP (920 μM, 4 eq.) recorded between 283 and 368 K at 1 K interval (heating rate 1 K min$^{-1}$, optical path 1 mm). The arrows indicate the evolution of the two main peaks ($A_{504nm}$, $A_{540nm}$). **b,** Ratio $A_{504nm}/A_{540nm}$ as a function of temperature for **PDI** (black points) and **p2-PDI** (red points) calculated as the average of four consecutive heating/cooling runs. Error bars show standard deviations. The solid lines correspond to the fitting with Supplementary Equation 1 representing a T-dependent isodesmic model, from which $\Delta H$ and $Tm$ where calculated (Supplementary Table 1). Adjusted $R^2 > 0.999$ for all the fits. **c,** Temperature dependent degree of aggregation, $\alpha(T)$, for **PDI** (black points) and **p2-PDI** (red points) corresponding to the curve in panel b, calculated by using Supplementary Equation 2. The solid black lines represent the theoretical $\alpha(T)$ for the isodesmic model calculated by using the values of $\Delta H$ and $Tm$ obtained from the fitting to the experimental data of panel b.

For both **PDI** and **p2-PDI** solutions, we recorded T-dependent UV-Vis spectra during two consecutive heating-cooling cycles between 283 and 368 K at 1 K intervals (heating/cooling rate 1 K $min^{-1}$). As an example, in Supplementary Figure 10a the absorption spectra recorded upon heating a 230 μM **PDI** solution are reported, which shows a progressive decrease of the ratio $A_{504nm}/A_{540nm}$ due to partial disassembly.[4] The ratio of the peak intensities $A_{504nm}/A_{540nm}$ (averaged over 4 consecutive heating/cooling runs) was plotted against T, as shown in Supplementary Figure 10b. The latter was fitted to a T-dependent isodesmic (equal-$K_{eq}$) model,[5,6] using the following equation,

$$Robs(T) = \frac{Rpol}{1+\exp[-0.908\Delta H \frac{T-Tm}{RTm^2}]} + \left(1-\frac{1}{1+\exp[-0.908\Delta H \frac{T-Tm}{RTm^2}]}\right) * Rmon \qquad 1$$

where $Robs(T) = A_{504nm}/A_{540nm}$ at a given T, *Rpol* corresponds to the ratio $(A_{504nm}/A_{540nm})_{\text{pol}}$ for the fully polymerized **PDI** or **p2-PDI** (that we got from the fitting), *Rmon* corresponds to the ratio $(A_{504nm}/A_{540nm})_{\text{mon}}$ for monomeric **PDI** or **p2-PDI** (set to 0.65), *ΔH* is the molar enthalpy related to the formation of the non-covalent interactions in the supramolecular polymerization, *Tm* is the (concentration dependent) melting temperature defined as the temperature for which the degree of aggregation α is 0.5 (see below), and *R* is the gas constant. By using Supplementary Equation 1, *ΔH* and *Tm* were determined for **PDI** and **p2-PDI** polymers.
The degree of aggregation, α(T), has been calculated using the following

$$\alpha(T) = \frac{Robs(T) - Rmon}{Rpol - Rmon} \qquad 2$$

and plotted against temperature in Supplementary Figure 10c.
From the degree of aggregation, the number averaged degree of polymerization (average stack length), *DPn* was calculated according to Supplementary Equation 3 and from the latter we could obtain the equilibrium constant, $K_{eq}$ (298 K), by using Supplementary Equation 4 (Supplementary Table 1).[5]

$$DP_N(T) = \frac{1}{\sqrt{1-\alpha(T)}} \qquad 3$$

$$Keq(T) = \frac{[(2DP_N - 1)^2 - 1]}{4C_T} \qquad 4$$

**Supplementary Table 1 |** Thermodynamic parameters for **PDI** and **p-2PDI** supramolecular polymerization in reaction buffer.

| **Sample** | ***ΔH*** (kJ $mol^{-1}$) | ***Tm*** (K) | ***DPn***[1] | $K_{eq}$ ($10^4$ $M^{-1}$), |
|---|---|---|---|---|
| **PDI** (230 μM) | -36.96±0.23 | 358.5 ± 0.11 | 2.77 | 2.14 |
| **p2-PDI** (230 μM) | -42.54 ± 0.2 | 368.9 ± 0.10 | 3.50 | 3.81 |

[1]Values determined at 298 K

## Additional AFM and TEM images

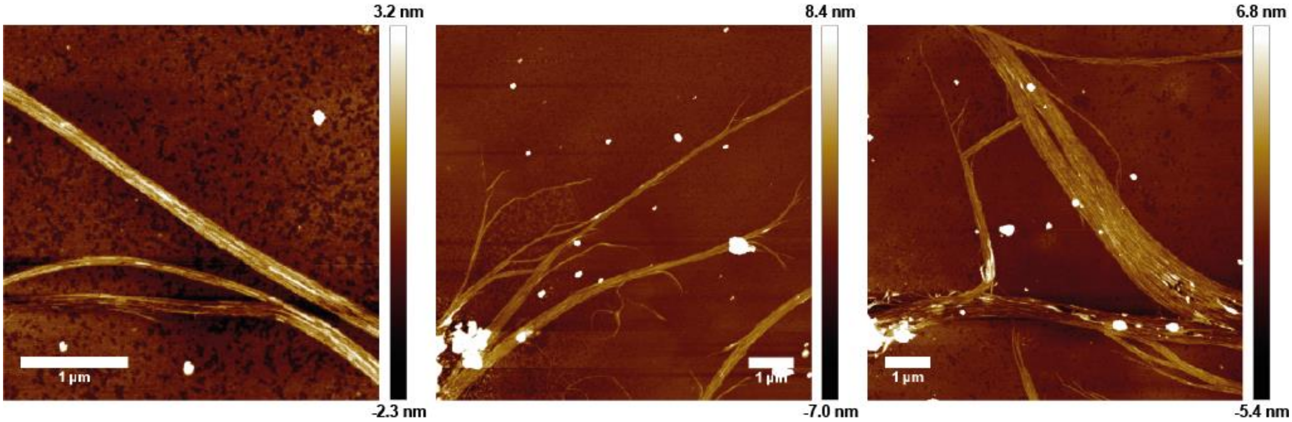


**Supplementary Figure 11 |** AFM images of **p2-PDI** aggregates on mica. The sample was prepared by diluting 100 times a 200 μM solution of **p2-PDI** in the reaction buffer with MQ water, followed by drop casting on mica.

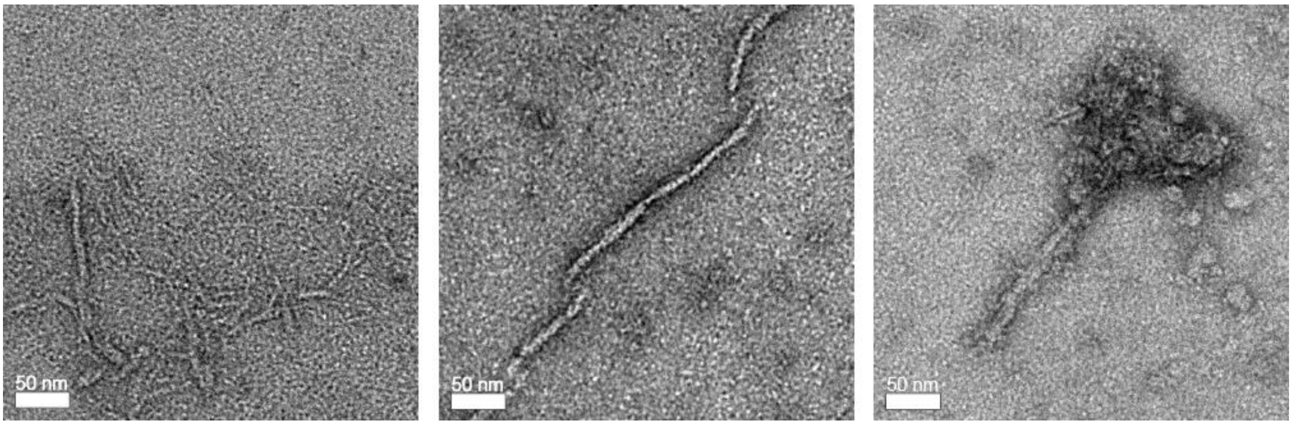


**Supplementary Figure 12 |** TEM images of **p2-PDI** assemblies adsorbed on a carbon film and negatively stained with a 2% uranyl acetate solution. The specimen was adsorbed for 90 min on a small piece of carbon partially floated off a mica sheet at the surface of a Teflon well containing 37 μL of a 80 μM **p2-PDI** solution in the reaction buffer. The carbon film was then transferred on an EM copper grid covered by a perforated carbon film (homemade holey grid).

## Effect of phosphorylation on supramolecular chirality studied by CD spectroscopy

### *Self-sorting experiments*

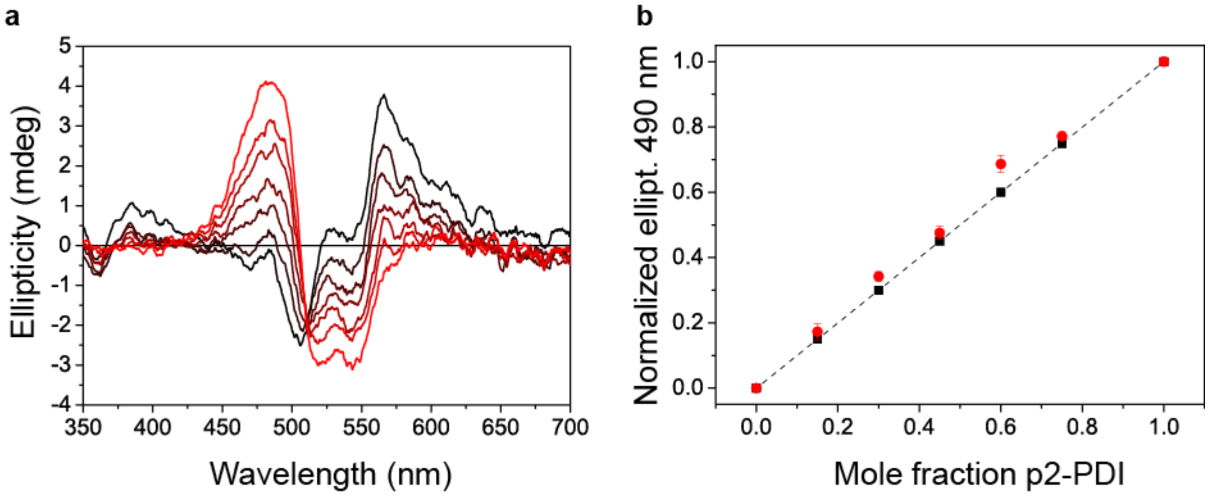


**Supplementary Figure 13 | a,** CD spectra of **PDI/p2-PDI** mixtures at different molar ratios from 0:1 (i.e. pure **PDI,** black spectrum) to 1:0 (i.e. pure **p2-PDI,** light red spectrum), at a total concentration (**PDI + p2-PDI**) of 200 μM in the reaction buffer (optical path 1mm). **b,** Normalized CD intensities at 490 nm, extracted by the CD spectra in panel a, plotted versus the corresponding molar fraction of **p2-PDI** (red circles). The data represent the average and standard deviation (error bars) calculated from two independent experiments. The dashed black line represents the theoretical values of the CD intensity at 490 as a function of the mixture composition for a completely self-sorted system (i.e. calculated as linear combination of the CD intensities of each component weighted on the corresponding molar fractions). The black squares highlight the theoretical value of the CD intensity at 490 for the specific compositions we investigated experimentally.

The samples for the self-sorting experiments were prepared by mixing different volumes of 200 μM **PDI** and **p2-PDI** solutions in the reaction buffer. **p2-PDI** was previously prepared by phosphorylation triggered by ATP (400 μM, 2 eq.) and PKA (0.065 μM). The mixed samples were incubated for 10 min at room temperature before the CD measurements. No changes of the spectra were observed for longer incubation times (e.g. overnight).

### *Phosphorylation followed by CD*

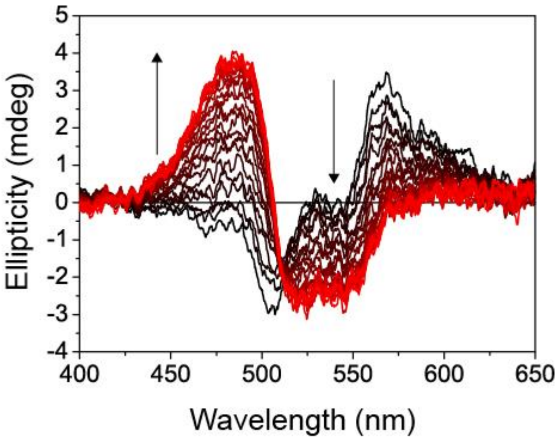


**Supplementary Figure 14 |** Time evolution of the CD spectrum (from black to red) during the phosphorylation triggered by addition of PKA (0.11 μM) and ATP (400 μM) to a 200 μM **PDI** solution in the reaction buffer at 25°C. Spectra recorded each 6 min, optical path 1 mm. The black arrows show the progressive inversion of the CD spectrum with the emergence of a positive Cotton effect at 475 nm and a negative Cotton effect at 530 nm.

## Mathematical modelling of the enzyme network

### *Description of the model*

A set of ordinary differential equation ODEs describing the network formed by the two enzymes Protein Kinase A (PKA) and Lambda Protein Phosphatase (λPP) was built using mass action kinetics. The enzyme PKA catalyses the transfer of the gamma phosphate of ATP to the LRRASL sequence (e.g. present in **PDI** and **p-PDI**) giving ADP and the phosphorylated peptide LRRApSL (i.e. **p-PDI** or **p2-PDI**) as products. PKA is modelled as "exiy" (Supplementary Figure 15), where "x" represents the binding site for ATP, "y" is that for LRRASL, and "i" is the binding site for non-competitive inhibitors.

λPP is a $Mn^{2+}$-dependent protein phosphatase that catalyses the cleavage of the phosphate group from phosphorylated serine (e.g. present in **p-PDI** or **p2-PDI**), giving non-phosphorylated peptide (i.e. **PDI**) and inorganic phosphate (Pi) as products. The latter involve a single-substrate mechanism, so we modelled λPP as "px" (Supplementary Figure 15), where "x" represents the binding site for the phosphopeptide.

The model takes into account:

1) A random sequential BiBi kinetic mechanism for PKA, in which both the substrates (ATP and LRRASL) bind, in no obligatory order, before the products are released.[7]
2) The presence of two (de)phosphorylation sites in each **PDI** molecule (Supplementary Figure 15)
3) The competitive (substrate) inhibition by LRRASL (present in **PDI** and **p-PDI**) that can bind to PKA's site for ATP (Supplementary Figure 15, green arrow in blue pocket, "ebiy", "eqiy", "ebqy", "ebry", "ebib", "ebiq", "eqqy", "eqry", "eqib", "eqiq", "ebqb", "ebqq", "ebrb", "ebrq", "eqqb", "eqqq", "eqrb", "ebrq").
4) The competitive (product) inhibition by ADP that can bind to the PKA's site for ATP[4] (Supplementary Figure 15, ADP in the blue pocket, "epiy", "epqy", "epry", "epib", "epiq", "epqb", "epqq", "eprb", "eprq").
5) The noncompetitive (product) inhibition by LRRApSL (present in **p-PDI** and **p2-PDI**) that can bind to PKA's site for non-competitive inhibitors[8] (Supplementary Figure 15, red arrow in red pocket, "exqy", "exry", "eaqy", "eary", "ebqy", "ebry", "epqy", "epry", "eqqy", "eqry", "exqb", "exqq", "exrb", "exrq", "eaqb", "eaqq", "earb", "earq", "ebqb", "ebqq", "ebrb", "ebrq", "epqb", "epqq", "eprb", "eprq", "eqqb", "eqqq", "eqrb", "eqrq").
6) A single-substrate mechanism, for λPP.
7) The competitive (product) inhibition by Pi that can bind to the λPP's site for the phosphopeptide,[9] (Supplementary Figure 15, "pk").
8) λPP does not feature any ATP hydrolytic activity.[10]

The latter results in a network of 55 chemical species (including free enzymes, substrates and products, and the various enzyme-substrate complexes) that are depicted in Supplementary Figure 15. In Supplementary Table 2 the binding equilibria and the conversion reactions, i.e. the edges of the network, are reported together with the corresponding rate equations based on mass-action kinetics. From the latter, the 55 ODEs describing the enzyme network (i.e. one for each species) were derived as reported in Supplementary Table 3. The set of ODEs were implemented in MATLAB and integrated numerically, using available solvers, in order to obtain the temporal

evolution of the concentration of all the species, starting from a set of initial conditions that correspond to the experimental conditions to simulate.

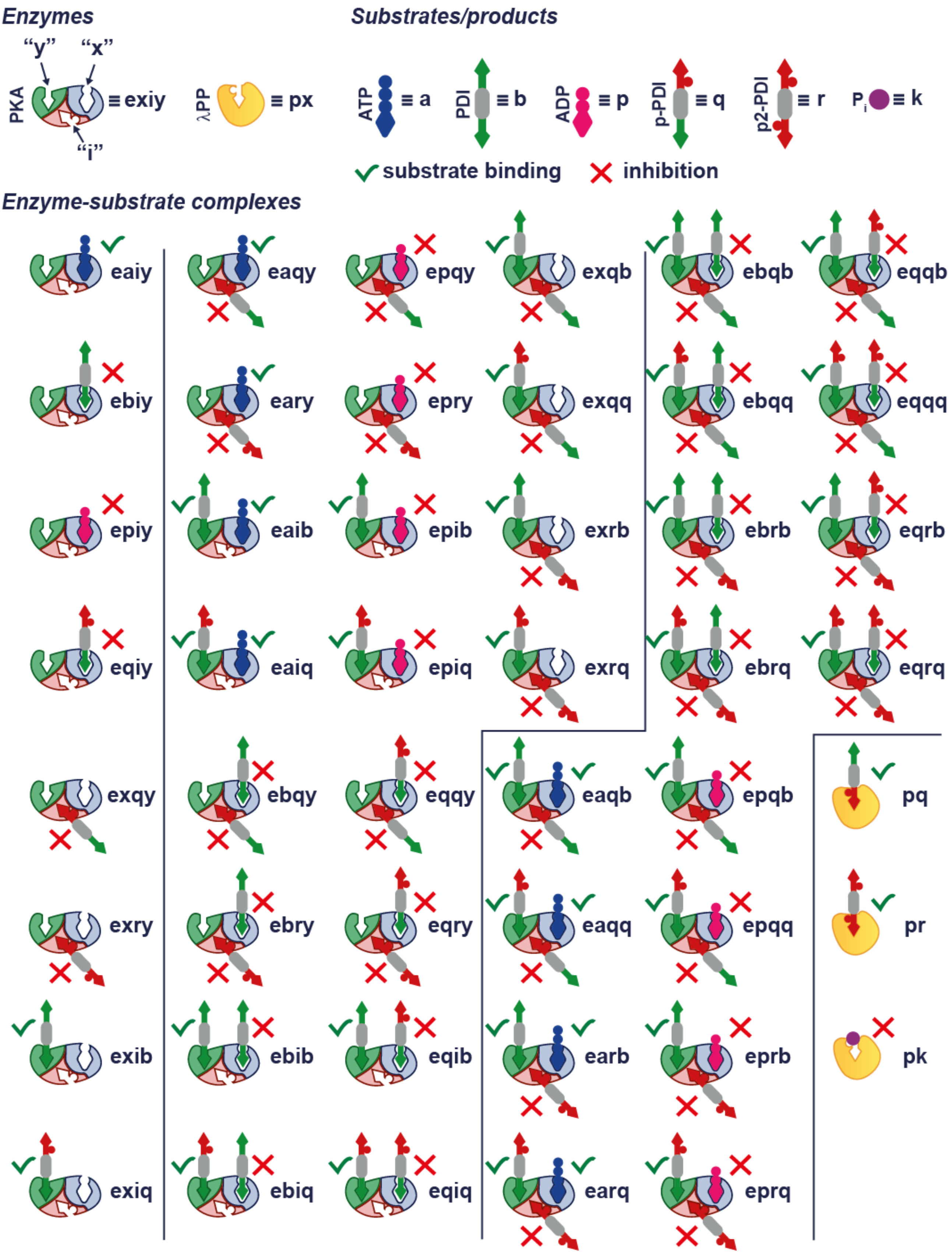


**Supplementary Figure 15 |** Overview of the species comprising the enzyme network (the nodes). The latter include: free enzymes, substrates and products, and the enzyme-substrate complexes. PKA has three binding sites: "x" for ATP

(blue), “y” is that for LRRASL, and “i” for non-competitive inhibitor LRRApSL (red). The PKA enzyme-substrate complexes can have one (first column), two (second column) or three (third column) binding sites occupied. The green ticks indicate the substrate binding, the red crosses indicate the binding of inhibitors.

**Supplementary Table 2 |** Overview of the involved binding and conversion reactions with the corresponding rate equations based on mass action kinetics.

| **Reaction** | **Rate equation** |
|---|---|
| ***Zero binding (PKA)*** | |
| 1) $exiy + a \rightleftarrows eaiy$ | $R1 = kiaf*c\_exiy*c\_a - kiar*c\_eaiy$ |
| 2) $exiy + b \rightleftarrows ebiy$ | $R2 = ksibf*c\_exiy*c\_b - ksibr*c\_ebiy$ |
| 3) $exiy + p \rightleftarrows epiy$ | $R3 = kpipf*c\_exiy*c\_p - kpipr*c\_epiy$ |
| 4) $exiy + q \rightleftarrows eqiy$ | $R4 = ksiqf*c\_exiy*c\_q - ksiqr*c\_eqiy$ |
| 5) $exiy + q \rightleftarrows exqy$ | $R5 = kncqf*c\_exiy*c\_q - kncqr*c\_exqy$ |
| 6) $exiy + r \rightleftarrows exry$ | $R6 = kncrf*c\_exiy*c\_r - kncrr*c\_exry$ |
| 7) $exiy + b \rightleftarrows exib$ | $R7 = kibf*c\_exiy*c\_b - kibr*c\_exib$ |
| 8) $exiy + q \rightleftarrows exiq$ | $R8 = kiqf*c\_exiy*c\_q - kiqr*c\_exiq$ |
| ***One binding (PKA)*** | |
| 9) $eaiy + q \rightleftarrows eaqy$ | $R9 = kncqf*c\_eaiy*c\_q - kncqr*c\_eaqy$ |
| 10) $eaiy + r \rightleftarrows eary$ | $R10 = kncrf*c\_eaiy*c\_r - kncrr*c\_eary$ |
| 11) $eaiy + b \rightleftarrows eaib$ | $R11 = kbf*c\_eaiy*c\_b - kbr*c\_eaib$ |
| 12) $eaiy + q \rightleftarrows eaiq$ | $R12 = kqf*c\_eaiy*c\_q - kqr*c\_eaiq$ |
| 13) $ebiy + q \rightleftarrows ebqy$ | $R13 = kncqf*c\_ebiy*c\_q - kncqr*c\_ebqy$ |
| 14) $ebiy + r \rightleftarrows ebry$ | $R14 = kncrf*c\_ebiy*c\_r - kncrr*c\_ebry$ |
| 15) $ebiy + b \rightleftarrows ebib$ | $R15 = kbf*c\_ebiy*c\_b - kbr*c\_ebib$ |
| 16) $ebiy + q \rightleftarrows ebiq$ | $R16 = kqf*c\_ebiy*c\_q - kqr*c\_ebiq$ |
| 17) $epiy + q \rightleftarrows epqy$ | $R17 = kncqf*c\_epiy*c\_q - kncqr*c\_epqy$ |
| 18) $epiy + r \rightleftarrows epry$ | $R18 = kncrf*c\_epiy*c\_r - kncrr*c\_epry$ |
| 19) $epiy + b \rightleftarrows epib$ | $R19 = kbf*c\_epiy*c\_b - kbr*c\_epib$ |
| 20) $epiy + q \rightleftarrows epiq$ | $R20 = kqf*c\_epiy*c\_q - kqr*c\_epiq$ |
| 21) $eqiy + q \rightleftarrows eqqy$ | $R21 = kncqf*c\_eqiy*c\_q - kncqr*c\_eqqy$ |
| 22) $eqiy + r \rightleftarrows eqry$ | $R22 = kncrf*c\_eqiy*c\_r - kncrr*c\_eqry$ |
| 23) $eqiy + b \rightleftarrows eqib$ | $R23 = kbf*c\_eqiy*c\_b - kbr*c\_eqib$ |
| 24) $eqiy + q \rightleftarrows eqiq$ | $R24 = kqf*c\_eqiy*c\_q - kqr*c\_eqiq$ |
| 25) $exqy + a \rightleftarrows eaqy$ | $R25 = kiaf*c\_exqy*c\_a - kiar*c\_eaqy$ |
| 26) $exqy + b \rightleftarrows ebqy$ | $R26 = ksibf*c\_exqy*c\_b - ksibr*c\_ebqy$ |
| 27) $exqy + p \rightleftarrows epqy$ | $R27 = kpipf*c\_exqy*c\_p - kpipr*c\_epqy$ |
| 28) $exqy + q \rightleftarrows eqqy$ | $R28 = ksiqf*c\_exqy*c\_q - ksiqr*c\_eqqy$ |
| 29) $exqy + b \rightleftarrows exqb$ | $R29 = kibf*c\_exqy*c\_b - kibr*c\_exqb$ |
| 30) $exqy + q \rightleftarrows exqq$ | $R30 = kiqf*c\_exqy*c\_q - kiqr*c\_exqq$ |
| 31) $exry + a \rightleftarrows eary$ | $R31 = kiaf*c\_exry*c\_a - kiar*c\_eary$ |
| 32) $exry + b \rightleftarrows ebry$ | $R32 = ksibf*c\_exry*c\_b - ksibr*c\_ebry$ |
| 33) $exry + p \rightleftarrows epry$ | $R33 = kpipf*c\_exry*c\_p - kpipr*c\_epry$ |
| 34) $exry + q \rightleftarrows eqry$ | $R34 = ksiqf*c\_exry*c\_q - ksiqr*c\_eqry$ |

| | |
|---|---|
| 35) $exry + b \rightleftarrows exrb$ | $R35 = kibf*c\_exry*c\_b - kibr*c\_exrb$ |
| 36) $exry + q \rightleftarrows exrq$ | $R36 = kiqf*c\_exry*c\_q - kiqr*c\_exrq$ |
| 37) $exib + a \rightleftarrows eaib$ | $R37 = kaf*c\_exib*c\_a - kar*c\_eaib$ |
| 38) $exib + b \rightleftarrows ebib$ | $R38 = ksibf*c\_exib*c\_b - ksibr*c\_ebib$ |
| 39) $exib + p \rightleftarrows epib$ | $R39 = kpipf*c\_exib*c\_p - kpipr*c\_epib$ |
| 40) $exib + q \rightleftarrows eqib$ | $R40 = ksiqf*c\_exib*c\_q - ksiqr*c\_eqib$ |
| 41) $exib + q \rightleftarrows exqb$ | $R41 = kncqf*c\_exib*c\_q - kncqr*c\_exqb$ |
| 42) $exib + r \rightleftarrows exrb$ | $R42 = kncrf*c\_exib*c\_r - kncrr*c\_exrb$ |
| 43) $exiq + a \rightleftarrows eaiq$ | $R43 = kaf*c\_exiq*c\_a - kar*c\_eaiq$ |
| 44) $exiq + b \rightleftarrows ebiq$ | $R44 = ksibf*c\_exiq*c\_b - ksibr*c\_ebiq$ |
| 45) $exiq + p \rightleftarrows epiq$ | $R45 = kpipf*c\_exiq*c\_p - kpipr*c\_epiq$ |
| 46) $exiq + q \rightleftarrows eqiq$ | $R46 = ksiqf*c\_exiq*c\_q - ksiqr*c\_eqiq$ |
| 47) $exiq + q \rightleftarrows exqq$ | $R47 = kncqf*c\_exiq*c\_q - kncqr*c\_exqq$ |
| 48) $exiq + r \rightleftarrows exrq$ | $R48 = kncrf*c\_exiq*c\_r - kncrr*c\_exrq$ |
| ***Two binding (PKA)*** | |
| 49) $eaqy + b \rightleftarrows eaqb$ | $R49 = kbf*c\_eaqy*c\_b - kbr*c\_eaqb$ |
| 50) $eaqy + q \rightleftarrows eaqq$ | $R50 = kqf*c\_eaqy*c\_q - kqr*c\_eaqq$ |
| 51) $eary + b \rightleftarrows earb$ | $R51 = kbf*c\_eary*c\_b - kbr*c\_earb$ |
| 52) $eary + q \rightleftarrows earq$ | $R52 = kqf*c\_eary*c\_q - kqr*c\_earq$ |
| 53) $ebqy + b \rightleftarrows ebqb$ | $R53 = kbf*c\_ebqy*c\_b - kbr*c\_ebqb$ |
| 54) $ebqy + q \rightleftarrows ebqq$ | $R54 = kqf*c\_ebqy*c\_q - kqr*c\_ebqq$ |
| 55) $ebry + b \rightleftarrows ebrb$ | $R55 = kbf*c\_ebry*c\_b - kbr*c\_ebrb$ |
| 56) $ebry + q \rightleftarrows ebrq$ | $R56 = kqf*c\_ebry*c\_q - kqr*c\_ebrq$ |
| 57) $epqy + b \rightleftarrows epqb$ | $R57 = kbf*c\_epqy*c\_b - kbr*c\_epqb$ |
| 58) $epqy + q \rightleftarrows epqq$ | $R58 = kqf*c\_epqy*c\_q - kqr*c\_epqq$ |
| 59) $epry + b \rightleftarrows eprb$ | $R59 = kbf*c\_epry*c\_b - kbr*c\_eprb$ |
| 60) $epry + q \rightleftarrows eprq$ | $R60 = kqf*c\_epry*c\_q - kqr*c\_eprq$ |
| 61) $eqqy + b \rightleftarrows eqqb$ | $R61 = kbf*c\_eqqy*c\_b - kbr*c\_eqqb$ |
| 62) $eqqy + q \rightleftarrows eqqq$ | $R62 = kqf*c\_eqqy*c\_q - kqr*c\_eqqq$ |
| 63) $eqry + b \rightleftarrows eqrb$ | $R63 = kbf*c\_eqry*c\_b - kbr*c\_eqrb$ |
| 64) $eqry + q \rightleftarrows eqrq$ | $R64 = kqf*c\_eqry*c\_q - kqr*c\_eqrq$ |
| 65) $eaib + q \rightleftarrows eaqb$ | $R65 = kncqf*c\_eaib*c\_q - kncqr*c\_eaqb$ |
| 66) $eaib + r \rightleftarrows earb$ | $R66 = kncrf*c\_eaib*c\_r - kncrr*c\_earb$ |
| 67) $eaiq + q \rightleftarrows eaqq$ | $R67 = kncqf*c\_eaiq*c\_q - kncqr*c\_eaqq$ |
| 68) $eaiq + r \rightleftarrows earq$ | $R68 = kncrf*c\_eaiq*c\_r - kncqr*c\_earq$ |
| 69) $ebib + q \rightleftarrows ebqb$ | $R69 = kncqf*c\_ebib*c\_q - kncqr*c\_ebqb$ |
| 70) $ebib + r \rightleftarrows ebrb$ | $R70 = kncrf*c\_ebib*c\_r - kncrr*c\_ebrb$ |
| 71) $ebiq + q \rightleftarrows ebqq$ | $R71 = kncqf*c\_ebiq*c\_q - kncqr*c\_ebqq$ |
| 72) $ebiq + r \rightleftarrows ebrq$ | $R72 = kncrf*c\_ebiq*c\_r - kncrr*c\_ebrq$ |
| 73) $epib + q \rightleftarrows epqb$ | $R73 = kncqf*c\_epib*c\_q - kncqr*c\_epqb$ |
| 74) $epib + r \rightleftarrows eprb$ | $R74 = kncrf*c\_epib*c\_r - kncrr*c\_eprb$ |

| | |
|---|---|
| 75) $epiq + q \rightleftarrows epqq$ | $R75 = kncqf*c\_epiq*c\_q - kncqr*c\_epqq$ |
| 76) $epiq + r \rightleftarrows eprq$ | $R76 = kncrf*c\_epiq*c\_r - kncrr*c\_eprq$ |
| 77) $eqib + q \rightleftarrows eqqb$ | $R77 = kncqf*c\_eqib*c\_q - kncqr*c\_eqqb$ |
| 78) $eqib + r \rightleftarrows eqrb$ | $R78 = kncrf*c\_eqib*c\_r - kncrr*c\_eqrb$ |
| 79) $eqiq + q \rightleftarrows eqqq$ | $R79 = kncqf*c\_eqiq*c\_q - kncqr*c\_eqqq$ |
| 80) $eqiq + r \rightleftarrows eqrq$ | $R80 = kncrf*c\_eqiq*c\_r - kncrr*c\_eqrq$ |
| 81) $exqb + a \rightleftarrows eaqb$ | $R81 = kaf*c\_exqb*c\_a - kar*c\_eaqb$ |
| 82) $exqb + b \rightleftarrows ebqb$ | $R82 = ksibf*c\_exqb*c\_b - ksibr*c\_ebqb$ |
| 83) $exqb + p \rightleftarrows epqb$ | $R83 = kpipf*c\_exqb*c\_p - kpipr*c\_epqb$ |
| 84) $exqb + q \rightleftarrows eqqb$ | $R84 = ksiqf*c\_exqb*c\_q - ksiqr*c\_eqqb$ |
| 85) $exqq + a \rightleftarrows eaqq$ | $R85 = kaf*c\_exqq*c\_a - kar*c\_eaqq$ |
| 86) $exqq + b \rightleftarrows ebqq$ | $R86 = ksibf*c\_exqq*c\_b - ksibr*c\_ebqq$ |
| 87) $exqq + p \rightleftarrows epqq$ | $R87 = kpipf*c\_exqq*c\_p - kpipr*c\_epqq$ |
| 88) $exqq + q \rightleftarrows eqqq$ | $R88 = ksiqf*c\_exqq*c\_q - ksiqr*c\_eqqq$ |
| 89) $exrb + a \rightleftarrows earb$ | $R89 = kaf*c\_exrb*c\_a - kar*c\_earb$ |
| 90) $exrb + b \rightleftarrows ebrb$ | $R90 = ksibf*c\_exrb*c\_b - ksibr*c\_ebrb$ |
| 91) $exrb + p \rightleftarrows eprb$ | $R91 = kpipf*c\_exrb*c\_p - kpipr*c\_eprb$ |
| 92) $exrb + q \rightleftarrows eqrb$ | $R92 = ksiqf*c\_exrb*c\_q - ksiqr*c\_eqrb$ |
| 93) $exrq + a \rightleftarrows earq$ | $R93 = kaf*c\_exrq*c\_a - kar*c\_earq$ |
| 94) $exrq + b \rightleftarrows ebrq$ | $R94 = ksibf*c\_exrq*c\_b - ksibr*c\_ebrq$ |
| 95) $exrq + p \rightleftarrows eprq$ | $R95 = kpipf*c\_exrq*c\_p - kpipr*c\_eprq$ |
| 96) $exrq + q \rightleftarrows eqrq$ | $R96 = ksiqf*c\_exrq*c\_q - ksiqr*c\_eqrq$ |
| ***Product formation (PKA)*** | |
| 97) $eaib \rightarrow exiy + p + q$ | $R97 = kcat*c\_eaib$ |
| 98) $eaiq \rightarrow exiy + p + r$ | $R98 = kcat*c\_eaiq$ |
| ***One binding (λPP)*** | |
| 99) $px + q \rightleftarrows pq$ | $R99 = kpqf*c\_px*c\_q - kpqr*c\_pq$ |
| 100) $px + r \rightleftarrows pr$ | $R100 = kprf*c\_px*c\_r - kprr*c\_pr$ |
| 101) $px + k \rightleftarrows pk$ | $R101 = kpikf*c\_px*c\_k - kpikr*c\_pk$ |
| ***Product formation (λPP)*** | |
| 102) $pq \rightarrow px + b + k$ | $R102 = kcatp*c\_pq$ |
| 103) $pr \rightarrow px + q + k$ | $R103 = kcatp*c\_pr$ |

**Supplementary Table 3 |** Overview of the differential equations describing the concentration versus time of the 55 species depicted in Supplementary Figure 15. The rates R1-R103 are defined in Supplementary Table 2.

| **Differential equations *(ODEs)*** |
|---|
| $\frac{d[a]}{dt} = -R1 - R25 - R31 - R37 - R43 - R81 - R85 - R89 - R93$ |
| $\frac{d[b]}{dt} = -R2 - R7 - R11 - R15 - R19 - R23 - R26 - R29 - R32 - R35 - R38 - R44 - R49 - R51 - R53$ |
| $-R55 - R57 - R59 - R61 - R63 - R82 - R86 - R90 - R94 + R102$ |
| $\frac{d[p]}{dt} = -R3 - R27 - R33 - R39 - R45 - R83 - R87 - R91 - R95 + R97 + R98$ |
| $\frac{d[q]}{dt} = -R4 - R5 - R8 - R9 - R12 - R13 - R16 - R17 - R20 - R21 - R24 - R28 - R30 - R34 - R36$ |
| $-R40 - R41 - R46 - R47 - R50 - R52 - R54 - R56 - R58 - R60 - R62 - R64 - R65 - R67 - R69$ |
| $-R71 - R73 - R75 - R77 - R79 - R84 - R88 - R92 - R96 + R97 - R99 + R103$ |
| $\frac{d[r]}{dt} = -R6 - R10 - R14 - R18 - R22 - R42 - R48 - R66 - R68 - R70 - R72 - R74 - R76 - R78$ |
| $-R80 + R98 - R100$ |
| $\frac{d[k]}{dt} = -R101 + R102 + R103$ |
| $\frac{d[exiy]}{dt} = -R1 - R2 - R3 - R4 - R5 - R6 - R7 - R8 + R97 + R98$ |
| $\frac{d[eaiy]}{dt} = +R1 - R9 - R10 - R11 - R12$ |
| $\frac{d[ebiy]}{dt} = +R2 - R13 - R14 - R15 - R16$ |
| $\frac{d[epiy]}{dt} = +R3 - R17 - R18 - R19 - R20$ |
| $\frac{d[eqiy]}{dt} = +R4 - R21 - R22 - R23 - R24$ |
| $\frac{d[exqy]}{dt} = +R5 - R25 - R26 - R27 - R28 - R29 - R30$ |
| $\frac{d[exry]}{dt} = +R6 - R31 - R32 - R33 - R34 - R35 - R36$ |
| $\frac{d[exib]}{dt} = +R7 - R37 - R38 - R39 - R40 - R41 - R42$ |
| $\frac{d[exiq]}{dt} = +R8 - R43 - R44 - R45 - R46 - R47 - R48$ |
| $\frac{d[eaqy]}{dt} = +R9 + R25 - R49 - R50$ |
| $\frac{d[eary]}{dt} = +R10 + R31 - R51 - R52$ |
| $\frac{d[eaib]}{dt} = +R11 + R37 - R65 - R66 - R97$ |
| $\frac{d[eaiq]}{dt} = +R12 + R43 - R67 - R68 - R98$ |
| $\frac{d[ebqy]}{dt} = +R13 + R26 - R53 - R54$ |

$$\frac{d[ebry]}{dt} = +R14 + R32 - R55 - R56$$

$$\frac{d[ebib]}{dt} = +R15 + R38 - R69 - R70$$

$$\frac{d[ebiq]}{dt} = +R16 + R44 - R71 - R72$$

$$\frac{d[epqy]}{dt} = +R17 + R27 - R57 - R58$$

$$\frac{d[epry]}{dt} = +R18 + R33 - R59 - R60$$

$$\frac{d[epib]}{dt} = +R19 + R39 - R73 - R74$$

$$\frac{d[epiq]}{dt} = +R20 + R45 - R75 - R76$$

$$\frac{d[eqqy]}{dt} = +R21 + R28 - R61 - R62$$

$$\frac{d[eqry]}{dt} = +R22 + R34 - R63 - R64$$

$$\frac{d[eqib]}{dt} = +R23 + R40 - R77 - R78$$

$$\frac{d[eqiq]}{dt} = +R24 + R46 - R79 - R80$$

$$\frac{d[exqb]}{dt} = +R29 + R41 - R81 - R82 - R83 - R84$$

$$\frac{d[exqq]}{dt} = +R30 + R47 - R85 - R86 - R87 - R88$$

$$\frac{d[exrb]}{dt} = +R35 + R42 - R89 - R90 - R91 - R92$$

$$\frac{d[exrq]}{dt} = +R36 + R48 - R93 - R94 - R95 - R96$$

$$\frac{d[eaqb]}{dt} = +R49 + R65 + R81$$

$$\frac{d[eaqq]}{dt} = +R50 + R67 + R85$$

$$\frac{d[earb]}{dt} = +R51 + R66 + R89$$

$$\frac{d[earq]}{dt} = +R52 + R68 + R93$$

$$\frac{d[ebqb]}{dt} = +R53 + R69 + R82$$

$$\frac{d[ebqq]}{dt} = +R54 + R71 + R86$$
$$\frac{d[ebrb]}{dt} = +R55 + R70 + R90$$
$$\frac{d[ebrq]}{dt} = +R56 + R72 + R94$$
$$\frac{d[epqb]}{dt} = +R57 + R73 + R83$$
$$\frac{d[epqq]}{dt} = +R58 + R75 + R87$$
$$\frac{d[eprb]}{dt} = +R59 + R74 + R91$$
$$\frac{d[eprq]}{dt} = +R60 + R76 + R95$$
$$\frac{d[eqqb]}{dt} = +R61 + R77 + R84$$
$$\frac{d[eqqq]}{dt} = +R62 + R79 + R88$$
$$\frac{d[eqrb]}{dt} = +R63 + R78 + R92$$
$$\frac{d[eqrq]}{dt} = +R64 + R80 + R96$$
$$\frac{d[px]}{dt} = -R99 - R100 - R101 + R102 + R103$$
$$\frac{d[pq]}{dt} = +R99 - R102$$
$$\frac{d[pr]}{dt} = +R100 - R103$$
$$\frac{d[pk]}{dt} = +R101$$

***Optimization of the rate constants by using experimental data***

The microscopic rate constants have been chosen at the beginning based on the values reported in literature for the rates of PKA,[11,12] as well as on the knowledge of the binding constants of substrates and inhibitors to PKA and λPP.[8,9] However, to match the model description to our experimental data, that we collected under different conditions (reaction buffer), we performed an optimization of the rate constants by a global minimization procedure. First we built a function in MATLAB whose output is the distance (summed square of residuals) between the concentration versus time profiles for the three species **PDI**, **p-PDI** and **p2-PDI** determined experimentally and those calculated by numerical integration for a given set of parameters (rate constants) and fixed initial conditions (the same as in the experiment). Secondly, we looked for the lowest minimum of the above nonlinear multivariable function (i.e. its global minimum) by using the Global Search option (with fmincon solver) within the MATLAB Global Optimization Toolbox. By inputting an

initial set of rate constants (Supplementary Table 4) as the starting point for the optimization problem, together with suitable upper and lower bounds, we got an optimized set of rate constants which correspond to the best match between the calculated and experimental temporal evolutions of **PDI**, **p-PDI** and **p2-PDI.** Remarkably, the Global Search algorithm was found to converge to practically the same solution even when starting quite far away from the minimum, e.g. with rate constants 3 of 4 orders of magnitude different from the optimized ones.

**Supplementary Table 4 |** Rate constants.

| ***Constants*** | ***Initial*** | ***Optimized*** | ***Units*** | ***Description*** |
|---|---|---|---|---|
| *kiaf* | $1.6 \cdot 10^5$ | $9.29 \cdot 10^6$ | $M^{-1}\ s^{-1}$ | Binding *a* to *exiy*, forward (*a* first to bind) |
| *kiar* | $2.0 \cdot 10^2$ | $6.84 \cdot 10^1$ | $s^{-1}$ | Binding *a* to *exiy*, reverse (*a* first to bind) |
| *kaf* | $5.0 \cdot 10^5$ | $1.57 \cdot 10^7$ | $M^{-1}\ s^{-1}$ | Binding *a* to *exiy*, forward (*a* second to bind) |
| *kar* | $3.5 \cdot 10^2$ | $4.29 \cdot 10^2$ | $s^{-1}$ | Binding *a* to *exiy*, reverse (*a* second to bind) |
| *kibf* | $5.9 \cdot 10^6$ | $1.27 \cdot 10^7$ | $M^{-1}\ s^{-1}$ | Binding *b* to *exiy*, forward (*b* first to bind) |
| *kibr* | $5.6 \cdot 10^2$ | $1.21 \cdot 10^1$ | $s^{-1}$ | Binding *b* to *exiy*, reverse (*b* first to bind) |
| *kbf* | $1.0 \cdot 10^7$ | $2.81 \cdot 10^6$ | $M^{-1}\ s^{-1}$ | Binding *b* to *exiy*, forward (*b* second to bind) |
| *kbr* | $3.5 \cdot 10^2$ | $3.83 \cdot 10^2$ | $s^{-1}$ | Binding *b* to *exiy*, reverse (*b* second to bind) |
| *kpqf* | $1.0 \cdot 10^6$ | $1.14 \cdot 10^3$ | $M^{-1}\ s^{-1}$ | Binding *q* to *px*, forward |
| *kpqr* | $1.0 \cdot 10^3$ | $2.30 \cdot 10^2$ | $s^{-1}$ | Binding *q* to *px*, reverse |
| *ksibf* | $1.0 \cdot 10^7$ | $1.57 \cdot 10^7$ | $M^{-1}\ s^{-1}$ | Subst. inhib. *b* binding to x-site of *exiy*, forward |
| *ksibr* | $1.0 \cdot 10^4$ | $1.99 \cdot 10^5$ | $s^{-1}$ | Subst. inhib. *b* binding to x-site of *exiy*, reverse |
| *ksiqf* | $5.9 \cdot 10^6$ | $3.66 \cdot 10^7$ | $M^{-1}\ s^{-1}$ | Subst. inhib. *q* binding to x-site of *exiy*, forward |
| *ksiqr* | $5.9 \cdot 10^3$ | $8.50 \cdot 10^4$ | $s^{-1}$ | Subst. inhib. *q* binding to x-site of *exiy*, reverse |
| *kncqf* | $1.0 \cdot 10^7$ | $3.31 \cdot 10^7$ | $M^{-1}\ s^{-1}$ | Noncomp. inhib. of *q* versus *exiy* forward |
| *kncqr* | $1.0 \cdot 10^4$ | $4.24 \cdot 10^4$ | $s^{-1}$ | Noncomp. inhib. of *q* versus *exiy* reverse |
| *kpipf* | $5.0 \cdot 10^5$ | $9.00 \cdot 10^7$ | $M^{-1}\ s^{-1}$ | Prod. inhib. *p* binding to x-site of *exiy*, forward |
| *kpipr* | $5.0 \cdot 10^2$ | $5.55 \cdot 10^1$ | $s^{-1}$ | Prod. inhib. *p* binding to x-site of *exiy*, reverse |
| *kpikf* | $1.0 \cdot 10^6$ | $7.93 \cdot 10^4$ | $M^{-1}\ s^{-1}$ | Prod. inhib. *k* binding to *px*, forward |
| *kpikr* | $1.0 \cdot 10^3$ | $2.29 \cdot 10^3$ | $s^{-1}$ | Prod. inhib. *k* binding to *px*, reverse |
| *kcat* | 3.0 | 2.93 | $s^{-1}$ | Reaction rate of kinase |
| *kcatp* | $1.0 \cdot 10^2$ | $4.75 \cdot 10^1$ | $s^{-1}$ | Reaction rate phosphatase |
| ***Constraints***[1] | | | | |
| *kiqf = kibf/2* | | | | Binding *q* to *exiy* (*q* first to bind), forward |
| *kiqr = kibr* | | | | Binding *q* to *exiy* (*q* first to bind), reverse |
| *kqf = kbf/2* | | | | Binding *q* to *exiy* (*q* second to bind), forward |
| *kqr = kbr* | | | | Binding *q* to *exiy* (*q* second to bind), reverse |
| *kprf = kpqf*2* | | | | Binding *r* to *px*, forward |
| *kprr = kpqr;* | | | | Binding *r* to *px*, reverse |
| *kncrf = kncqf*2* | | | | Noncomp. inhib. of *r* versus *exiy* forward |
| *kncrr = kncqr;* | | | | Noncomp. inhib. of *r* versus *exiy* reverse |

[1]Based on statistical arguments due to the presence of one or two sites of (de)phosphorylation.

### *Analysis of the reaction rates*

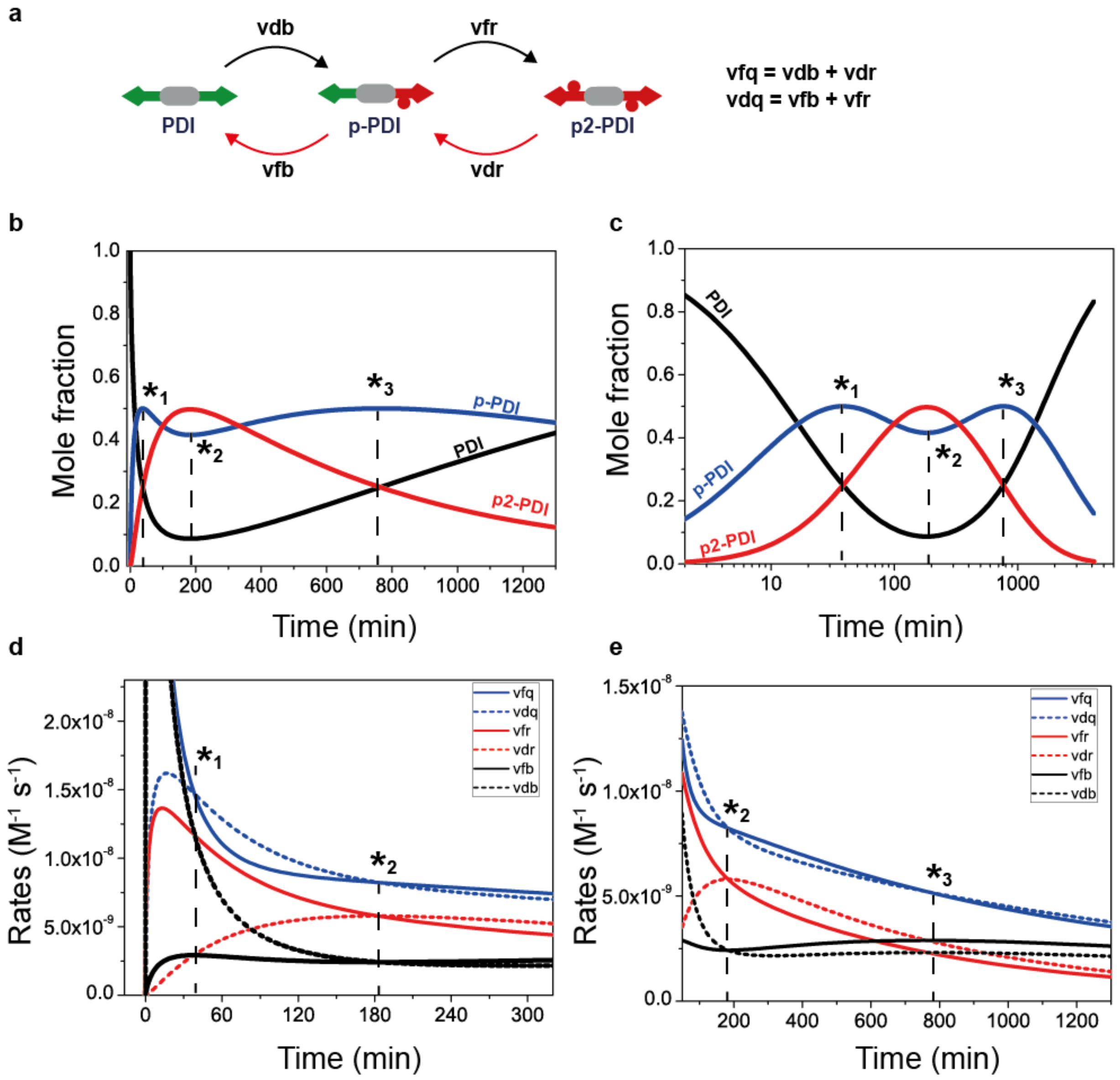


**Supplementary Figure 16 | a,** Rates of formation and disappearence of **PDI**, **p-PDI**, and **p2-PDI,** due to their interconversion. **b,** Simulation of the transient assembly reported in Fig 3a of the main text, with **PDI** = 100 μM, ATP = 1 mM, PKA = 0.13 μM, and λPP = 0.3 μM, as initial concentraions and using the optimized kinetic constants reported in Supplementary Table 4. **c,** The same plot as panel (b) reported in semilog scale to better appreciate the maxima. **d,** Change with time of the rates defined in panel (a) during the transient assembly reported in panel (b): zoom at shorter times; **e,** Same data as (d) but zoomed at longer times.

From the mathematical model (Supplementary Figure 15) it is possible to calculate the rates of formation and disappearence of the three species, **PDI**, **p-PDI**, **p2-PDI**, due to their interconversion (Supplementary Figure 16a), where vdb = R97; vfr = R98; vdr = R103; vfb = R102; vfq = R97+R103; vdq = R98+R102, as defined in Supplementary Table 2.

We used these values to rationalize the behaviour observed for monophosphorylated **p-PDI**, during the transient assembly, reported in Fig. 3a of the main text, with two maxima observed at around 37 and 180 min (Supplementary Figure 16 and 17).

At the beginning the rate of **PDI** disappearance (vdb) due to the first phosphorylation PDI→p-PDI is the highest (dashed black), which yields a fast accumulation of **p-PDI** (high vfq, blue solid). The latter occurs until the first maximum of **p-PDI** concentration (37 min, *1) is reached, at which the velocity of **p-PDI** disappearance (vdq, dashed blue) matches that of its formation (vfq, solid blue). From this point, and until 183 min (*2) the concentration of **p-PDI** starts to decrease (vdq > vdf), which is mainly due to the fact that the rate of formation of **p2-PDI** overcame that of disappearance of **PDI** (vfr > vdb) or, in other words, the phosphorylation p-PDI→p2-PDI is faster than PDI→ p-PDI. Note that so far the concentration profiles are mainly dominated by the reactions of PKA. Beyond 180 min the concentration of **p-PDI** starts to increase again (vdf > vdq ) until a new maximum at around 750 min (*3). The latter occurs because the disappearance of **p2-PDI** becomes faster than its formation (vdr < vfr), i.e. the dephosphorylation p2-PDI→p-PDI is faster than the phosphorylation p-PDI→p2-PDI. Note that in this time interval, also p-PDI→PDI is faster than PDI→p-PDI, (vfb > vdb) that would consume **p-PDI**, but the absolute value of these rates are lower than the former. Finally beyond 750 min, the concentration of **p-PDI** starts to slowly decay, which is due to the fact that the dephosphorylation p-PDI→ PDI become faster than the dephosphorylation p2-PDI→p-PDI (vfb > vdr), and remains the fastest reaction until the end of the transient assembly.

***Simulation arrays of enzymatic ODE model***

In addition to performing a global optimization to obtain more accurate rate constants (cf. Fig. 3a in the main text), we performed simulations over a wide range of PKA ($10^{-3}$ to $10^{-8}$ M), λPP ($10^{-3}$ to $10^{-8}$ M), and ATP ($10^{-4}$ to $10^{-0.5}$ M) concentrations (Supplementary Figure 17). In this way, we use the enzymatic ODE model (see above) to establish the time and amplitude of a transient excursion in **p2-PDI** (in a closed system). In other words, depending on the concentrations we can determine at what time the most **p2-PDI** is present in the solution. Starting from the experimental conditions (cf. Fig. 3a) we obtain a transient change in **p2-PDI** concentration with a maximum fraction of **p2-PDI** of ~0.5 (i.e., half of all initial **PDI** has been di-phosphorylated), occurring at ~$10^4$ s (=183 min.), shown in inset #1 (Supplementary Figure 17). If the concentration of λPP is increased significantly, the amplitude of the excursion is lowered (~0.01, Supplementary Figure 17a,b, #2), but it happens much faster (74 s, Supplementary Figure 17c,d, #2). To get a fast and large transient change, we can increase all concentrations (#3), leading to a max. frac. **p2-PDI** of ~0.93 after only 127 s. In short, these simulations show that both the amplitude and timescale of the transient change can be changed at will. The (competitive and non-competitive) inhibition pathways in the system (see above) do make the timescale of the excursion (Supplementary Figure 17c,d,) quite non-trivial.

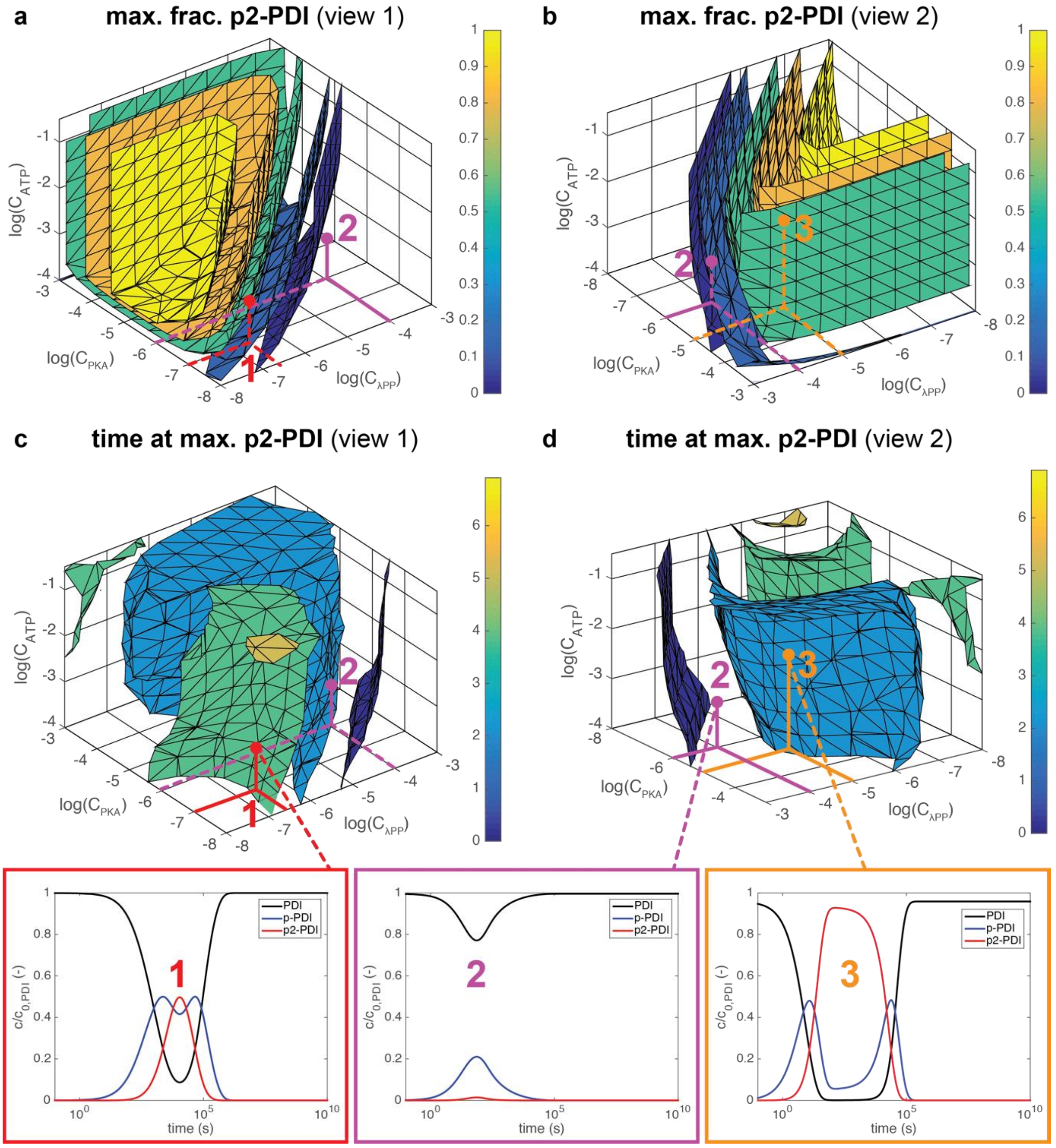


**Supplementary Figure 17 |** Numerical simulations of enzymatic ODE model (100 μM **PDI**) as a function of concentration of PKA, λPP, and ATP. Two characteristics are plotted: the maximum fraction of **p2-PDI** reached during the transient change (i.e., concentration **p2-PDI** divided by the initial **PDI** concentration), and ii) the time at which the maximum fraction of **p2-PDI** occurs. **a,** Max. frac. **p2-PDI** (linear color scale: blue is 0, yellow is 1). **b,** The same plot as panel **a** rotated 180° about the z-axis. **c,** Time at max. **p2-PDI** (logarithmic color scale: blue is 1 s, yellow is $10^7$ s). **d,** The same plot as panel **c** rotated 180° about the z-axis. Insets (1–3) show the concentrations of **PDI**, **p-PDI**, and **p2-PDI** at different PKA, λPP, and ATP concentrations. Inset 1 (cf. Fig. 3a of the main text): PKA (0.13 μM), λPP (0.3 μM), ATP (1 mM). Inset 2: PKA (1 μM), λPP (0.1 mM), ATP (1 mM), showing only a very small fraction of **p2-PDI** at the maximum. Inset 3: PKA (10 μM), λPP (10 μM), ATP (10 mM), resulting in a very high transient change in **p2-PDI** fraction.

## Effect of waste on multiple transient experiments.

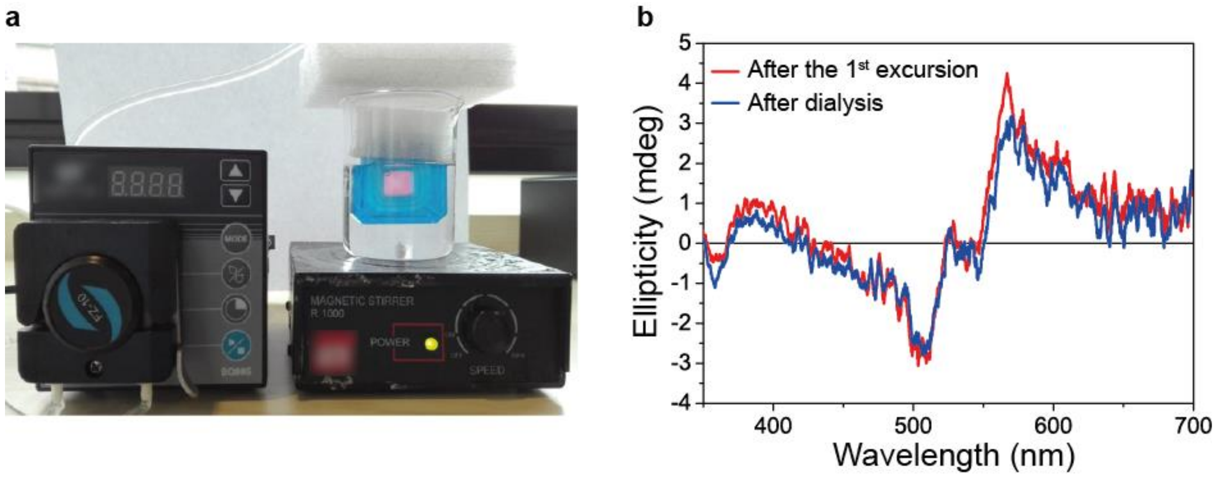


**Supplementary Figure 18 | a,** Dialysis to get rid of the waste (ADP + Pi). The pink solution (300 µL) coming from the ATP refuelling experiment at 2160 min (see Fig. 3c, main text), which contained only **p2-PDI** (as checked by LC-MS), was put in a Slide-A-Lyzer™ dialysis cassette with a molecular weight cut-off, MWCO = 2 kD (i.e. permeable only to ATP, ADP amd Pi), and dialyzed against fresh reaction buffer overnight, continuously replenished by a peristaltic pump. **b,** CD spectra of the above solution after overnight dialysis (blue line) as compared with the CD spectrum of the same solution recorded just after the first transient self-assembly (red line), i.e. recorded at 750 min in the experiment reported in Fig. 3b of the main text. The latter clearly shows that by removing waste the system is able to relax completely back to the initial non-phosphorylate state, **PDI** (as verified also by LC-MS).

## LC-MS spectra at the NESSs

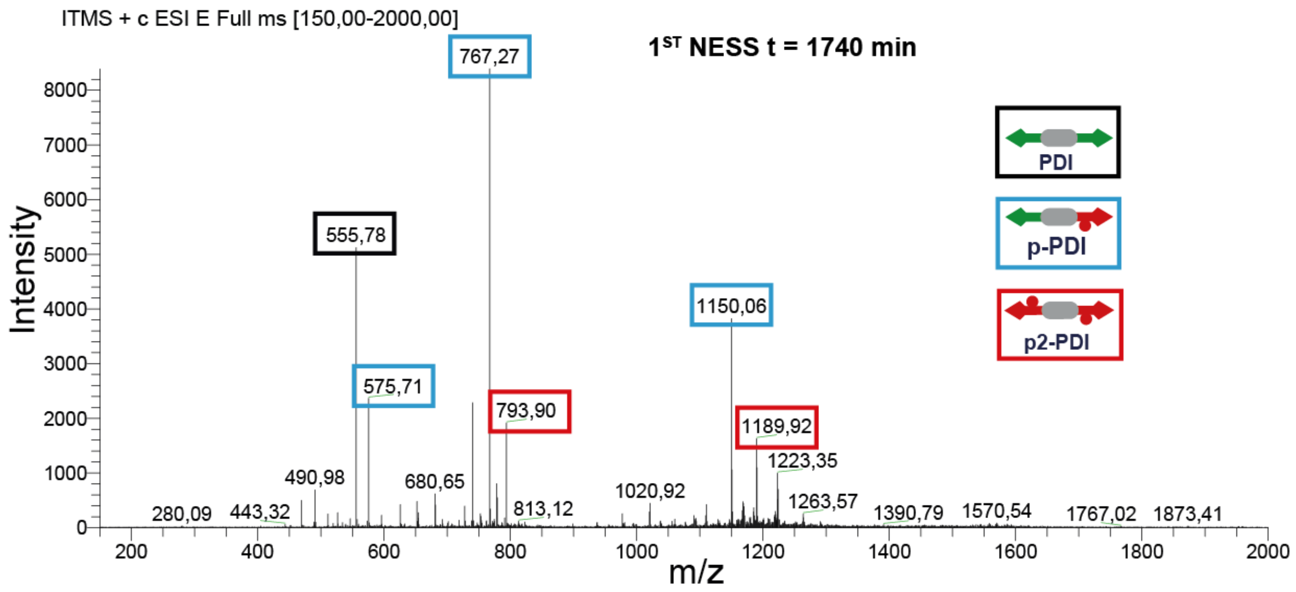


**Supplementary Figure 19 |** LC-MS spectrum measured along the first NESS obtained by flowing continuously 1 mM ATP (first plateau in Fig. 4c, sampled at t = 1740 min).

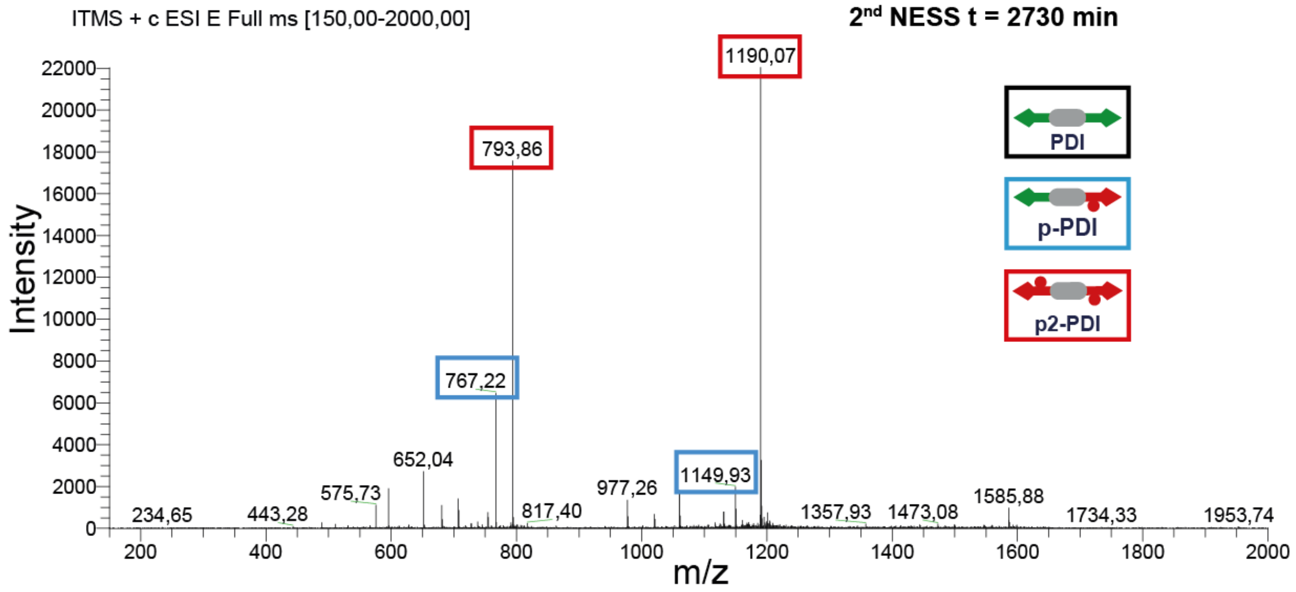


**Supplementary Figure 20 |** LC-MS spectrum measured along the second NESS obtained by flowing 2.5 mM ATP (second plateau in Fig. 4c, sampled at t = 2730 min).

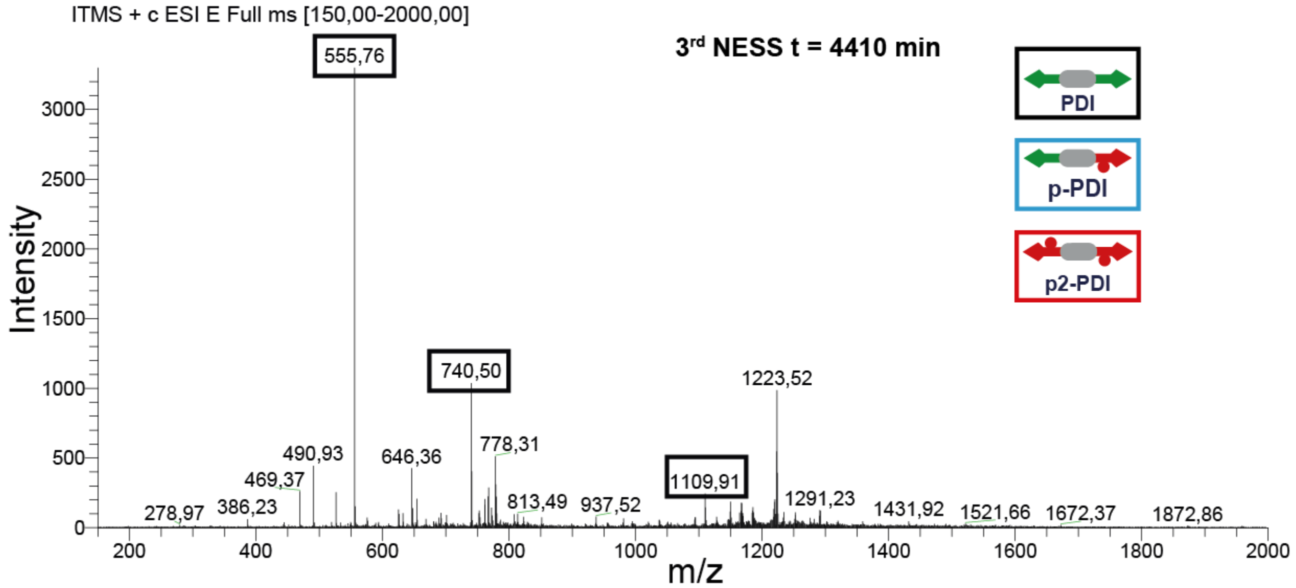


**Supplementary Figure 21 |** LC-MS spectrum measured along the third NESS obtained by flowing only buffer without ATP (third plateau in Fig. 4c, sampled at t = 4410 min).

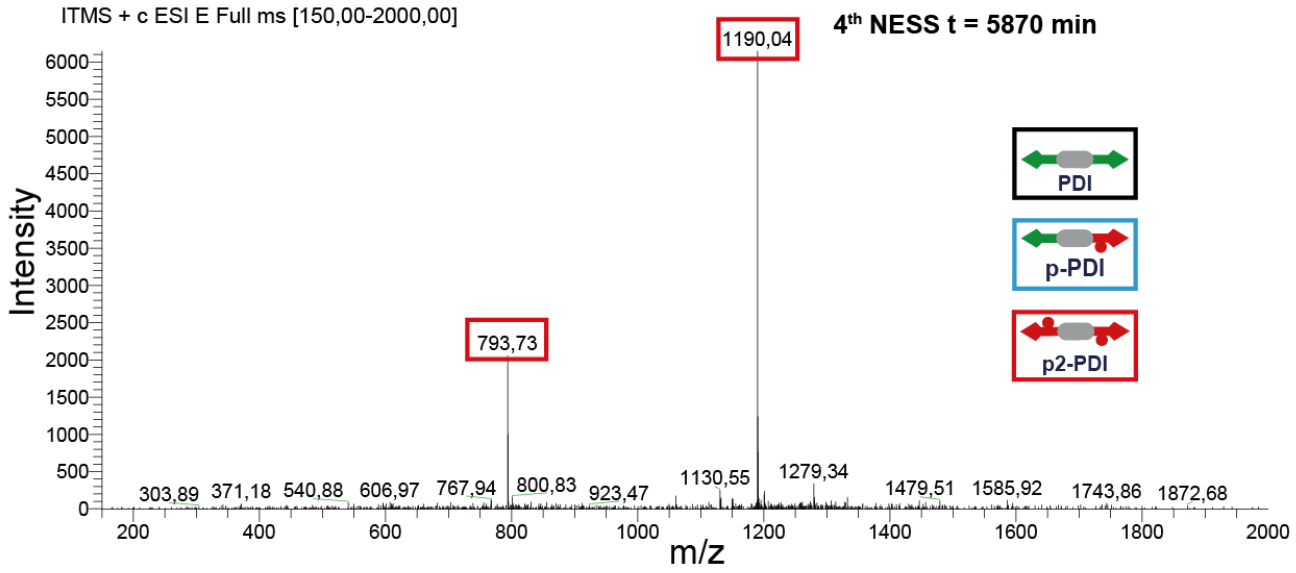


**Supplementary Figure 22 |** LC-MS spectrum measured along the fourth NESS obtained by flowing 5 mM ATP (fourth plateau in Fig. 4c, sampled at t = 5870 min).

## Supplementary References


1. Isidro-Llobet, A., Alvarez, M. & Albericio, F. Amino acid-protecting groups. *Chem. Rev.* **109,** 2455–2504 (2009).
2. Scherman, O. A., Ligthart, G. B. W. L., Ohkawa, H., Sijbesma, R. P. & Meijer, E. W. Olefin metathesis and quadruple hydrogen bonding: A powerful combination in multistep supramolecular synthesis. *Proc. Natl. Acad. Sci.* **103,** 11850–11855 (2006).
3. Görl, D., Zhang, X. & Würthner, F. Molecular Assemblies of Perylene Bisimide Dyes in Water. *Angew. Chem. Int. Ed.* **51,** 6328–6348 (2012).
4. Görl, D. & Würthner, F. Entropically Driven Self-Assembly of Bolaamphiphilic Perylene Dyes in Water. *Angew. Chem.* **128,** 12273–12277 (2016).
5. Smulders, M. M. J. *et al.* How to Distinguish Isodesmic from Cooperative Supramolecular Polymerisation. *Chem. – Eur. J.* **16,** 362–367 (2010).
6. De Greef, T. F. A. *et al.* Supramolecular Polymerization. *Chem. Rev.* **109,** 5687–5754 (2009).
7. Adams, J. A. Kinetic and Catalytic Mechanisms of Protein Kinases. *Chem. Rev.* **101,** 2271–2290 (2001).
8. Whitehouse, S., Feramisco, J. R., Casnellie, J. E., Krebs, E. G. & Walsh, D. A. Studies on the kinetic mechanism of the catalytic subunit of the cAMP-dependent protein kinase. *J. Biol. Chem.* **258,** 3693–3701 (1983).
9. Reiter, N. J., White, D. J. & Rusnak, F. Inhibition of Bacteriophage λ Protein Phosphatase by Organic and Oxoanion Inhibitors. *Biochemistry (Mosc.)* **41,** 1051–1059 (2002).
10. Zhuo, S., Clemens, J. C., Hakes, D. J., Barford, D. & Dixon, J. E. Expression, purification, crystallization, and biochemical characterization of a recombinant protein phosphatase. *J. Biol. Chem.* **268,** 17754–17761 (1993).
11. Sims, P. C. *et al.* Electronic Measurements of Single-Molecule Catalysis by cAMP-Dependent Protein Kinase A. *J. Am. Chem. Soc.* **135,** 7861–7868 (2013).
12. Lew, J., Taylor, S. S. & Adams, J. A. Identification of a partially rate-determining step in the catalytic mechanism of cAMP-dependent protein kinase: A transient kinetic study using stopped-flow fluorescence spectroscopy. *Biochemistry (Mosc.)* **36,** 6717–6724 (1997).
